# Genetic adaptation of microbial populations present in high-intensity catfish production systems with therapeutic oxytetracycline treatment

**DOI:** 10.1038/s41598-017-17640-3

**Published:** 2017-12-13

**Authors:** Qifan Zeng, Xiangli Tian, Luxin Wang

**Affiliations:** 1 Food Microbiology and Safety Lab, Department of Animal Sciences, Auburn University, Auburn, Alabama USA; 2 The Key Laboratory of Mariculture, Ocean University of China, Qingdao, China

## Abstract

Microbial communities that are present in aquaculture production systems play significant roles in degrading organic matter, controlling diseases, and formation of antibiotic resistance. It is important to understand the diversity and abundance of microbial communities and their genetic adaptations associated with environmental physical and chemical changes. Here we collected water and sediment samples from a high-intensity catfish production system and its original water reservoir. The metagenomic analysis showed that Proteobacteria, Actinobacteria, Bacteroidetes, Cyanobacteria, and Firmicutes were the top five phyla identified from all samples. The aquaculture production system significantly changed the structure of aquatic microbial populations. Substantial changes were also observed in SNP patterns among four sample types. The gene-specific sweep was found to be more common than genome-wide sweep. The selective sweep analysis revealed that 21 antibiotic resistant (AR) genes were under selection, with most belonging to antibiotic efflux pathways. Over 200 AR gene gains and losses were determined by changes in gene frequencies. Most of the AR genes were characterized as ABC efflux pumps, RND efflux pumps, and tetracycline MFS efflux pumps. Results of this study suggested that aquaculture waste, especially waste containing therapeutic antibiotics, has a significant impact on microbial population structures and their genetic structures.

## Introduction

Aquaculture has been a fast-growing industry in recent years because of dramatic increases in fish and seafood demand worldwide. As a result, high-intensity production systems, such as in-pond raceways (IPRS), have received increasing attention. Such production systems are developed with the dual goals of reducing production costs and increasing production yield^1^. Unfortunately, concerns about the impact of aquaculture wastes on the environment and the ecosystem have also increased. Aquaculture production wastes, such as ammonia and phosphorus, are the result of the excretion and decomposition of unconsumed feed^2–4^. In addition, antibiotic use in aquaculture has induced the emergence and dissemination of diverse antibiotic-resistant genes and microorganisms. According to Cabello *et al*., approximately 80% of antimicrobials used in aquaculture enter the environment with intact activity, with the ability to exert selection for resistant bacteria in microbial populations with diverse insensitivity^5^. Alarmingly, antibiotic-resistant fish- and human- pathogens, such as *Aeromonas* ^6,7^, *Vibrio* ^8^, and *Salmonella* ^9,10^, have been isolated from aquaculture operations.

Microbial communities consist of genetically and ecologically distinct groups. They are important constituents of the aquatic ecosystem. Microorganisms present in aquaculture production systems play significant roles in nutrient recycling, degradation of organic matter, and treatment and control of disease^11^. The abundance and diversity of microbial communities as well as their genetic structures are directly associated with the physical and chemical properties of the aquaculture production environment^11,12^. These physical and chemical properties are largely determined by production practices, such as feed frequencies and the application of antibiotics.

Microbial communities respond rapidly to changes in their immediate environment, and these changes may be subtle and may manifest themselves as activation or inactivation of different metabolic pathways^11,13^. Aquacultural microbiomes are likely to be system-specific. In recent decades, monitoring and manipulating microbial communities in aquaculture environments have shown great potential. For example, due to the increasing concern regarding antibiotic-resistant bacteria and risks, the addition of probiotics to feed for disease prevention or treatment is proposed as an alternative way to treat diseases without the use of antibiotics. Unfortunately, the lack of knowledge about the ecology of the microbiomes present in different production systems currently hampers the successful management of aquaculture microbial communities^11^. Studies that can better illustrate interactions between microbial communities and complex aquaculture production systems are still needed.

The development of metagenomics has made the study of microbial community structures in a given environment possible. It has been found that bacterial communities consist of closely related organisms and display cohesive ecological associations that distinguish them from each other^14–16^. Comparisons of the sequence polymorphism within a population or the divergence between populations can be used to identify potential genetic loci affected by selection pressures. These analyses have helped in estimating levels of recombination and mutation occurring within or between different sequence clusters^17,18^. More importantly, such analyses have also provided the foundation for manipulating and engineering microbial populations in a given environment^19^.

One major constraint in aquaculture is disease outbreaks, bacterial fish pathogens are considered the most important infectious microbes^20^. The development of antibiotic resistance is outpacing the discovery and development of new antibiotics, and the fact that certain bacterial infections are becoming untreatable has made evaluation of the therapeutic usage of antibiotics an urgent need. Such evaluation should include analysis of the diversity and abundance of microbial populations as well as changes and adaptations in their genetic structures. In bacteria, genetic adaptations to environmental changes are achieved through the selection of advantageous mutations and the horizontal transfer of beneficial genes^21^. For example, based on the mutant selective window hypothesis, directional selection usually occurs at antimicrobial concentrations between the minimal inhibitory concentration (MIC) of the susceptible bacteria and that of the resistant population^22,23^. Other studies suggest that antimicrobial concentrations lower than MIC also can enrich resistant bacteria from populations with minute differences in insensitivity to antibiotics^24,25^. Because of the differences seen between previous findings and theories, adaptive laboratory evolution experiments have been conducted in order to better determine the potential selection regime of microbial resistance^26,27^. Unfortunately, most of these studies were limited to one or a few bacterial species cultured in the laboratory environment and did not reflect the dynamic evolutionary processes that occur in a real production system. Given that antibiotics are used in aquaculture only for disease treatment and require a veterinary prescription, an evaluation focusing on the therapeutic usage of antibiotics in a real aquaculture production system is needed.

In summary, although improving the production efficiency of current aquaculture production systems through manipulating and engineering microbial populations is promising, information on the genetic adaptation of microbial populations present in different complex production systems is still missing. The goal of this study is to address this knowledge gap by providing an in-depth evaluation of the genetic adaptations that occur in a high-intensity production system (the in-pond raceways system) and the interactions between bacterial populations and their ecological niches. By conducting metagenomics sequencing, genetic factors underlying the heterogeneity in antimicrobial resistance and metabolic alterations were also investigated.

## Results

### Microbial community analysis

A total of 278.5 million reads with the average read length of 100 bp were generated from all sediment and water samples. After trimming, a total of 270.2 million filtered reads (~97%) were kept for further analysis (Supplementary Table S1). A total of 145.8 million and 124.3 million filtered reads were obtained from the sediment and water samples respectively.

Kaiju and Phylosift software were used to generate the taxonomic profile. In total, 21.78 million filtered reads of the “treatment” sediment dataset were assigned to the superkingdom level, accounting for 35.2% of the total reads. For the “treatment” water samples, 20.75 million reads (28.5% of the total reads) were matched to reference sequences at the superkingdom rank. For the water and sediment samples collected from the control system, 29.88 M (35.2%) and 19.04 M (36.9%) reads were annotated at the rank of superkingdom respectively.

**Figure 1:**
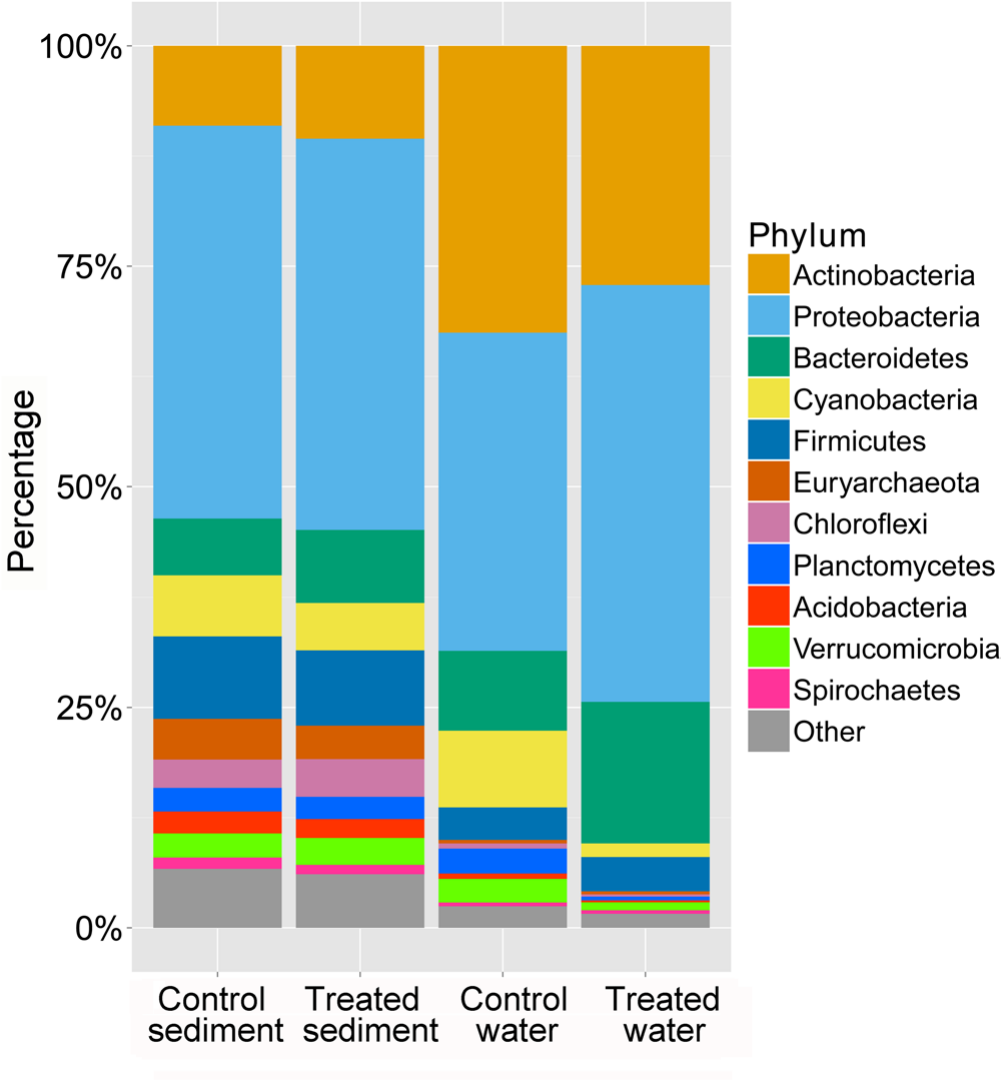
Microbiome composition at the phylum level of four different sample types (water samples collected from the IPRS system “treated water,” sediment samples collected from the IPRS system “treated sediment,” water samples collected from the control pond “control water,” and sediment samples collected from the control pond “control sediment”). The top 11 phyla (accounts for over 90% of matches at the phylum level) were reported for each sample, and all other phyla were grouped into “Other.”

Bacteria accounted for over 94% of the identified sequences in all the samples (Supplementary Fig. S1). Proteobacteria, Actinobacteria, Bacteroidetes, Cyanobacteria, and Firmicutes were the top five phyla identified from all four types of samples (Fig. 1). These five phyla accounted for 76% and 77% of the total bacterial populations present in the treatment and control sediment samples collected. For the treatment and control water samples, these five phyla accounted for more than 90% and 95% of the total bacteria identified.

Differences were also observed in the relative abundance of archaeal microorganisms and viruses when comparing sediment samples with water samples. Archaeal microorganisms were more abundant in sediment than in water samples (5% and 4% in “control” and “treatment” sediments, 0.5% and 0.4% in “control” and “treatment” water respectively), with Euryarchaeota as the most abundant phylum (5% in “control” sediment and 4% in “treatment” sediment). Viral communities were observed at higher levels in water samples than in sediment samples (0.2% and 0.4% in “control” and “treatment” sediment, 3% and 6% in “control” and “treatment” water), with Caudovirales being the most abundant order (2.5% in “control” water and 5.3% in “treatment” water).

The community composition of the four types of samples collected exhibited significant differences at the genus level. The average Shannon diversity index is 7.50 in the sediment samples and 6.93 in the water samples, indicating that sediment samples have a higher level of diversity compared to water samples. The principal component analysis revealed drastic differences in microbial populations present in the water and sediment samples. Sample types (sediment vs. water) explained 85.5% of the variance observed among the genomes sequenced, while the “treatment” and “control” factors explained only 13.2% of the abundance variabilities.

**Figure 2:**
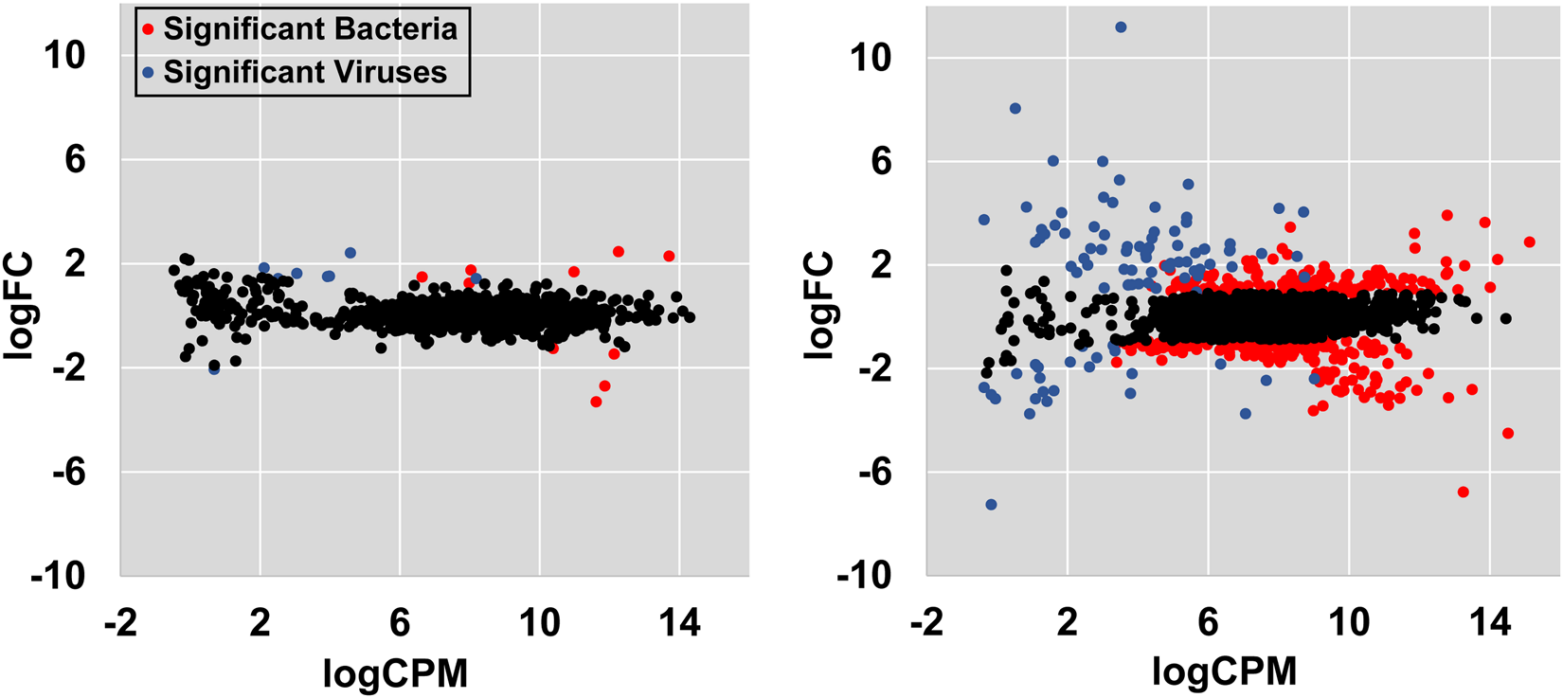
Comparisons of bacterial genera abundance between treatment and control samples (Left, sediment samples; Right, water samples).

Pairwise comparisons were conducted in order to investigate the change in abundance of the different genera present in water and sediment samples collected from both systems (treatment vs. control). For water samples, a total of 437 genera were identified with different abundance between the treatment and the control, including 339 bacterial and archaeal genera and 98 viral genera (Fig. 2, Supplementary Table S2). Bacteria that were significantly enriched in the water samples collected from IPRS belonged to 7 phyla. Proteobacteria remained the leading phyla, accounting for 53.3% of the genera identified (Supplementary Dataset S1). In contrast, only 18 genera showed significantly different abundance between the treated sediment samples and the control sediment samples, including 10 bacterial and archaeal genera and 8 viral genera (Supplementary Dataset S2).

### De novo metagenome assembly and phylogenetic assignment

Bacterial genomes were reconstructed from a combined assembly of metagenomic sequences. A total of 932,714 contigs with N50 size of 1,930 bp were assembled from the pooled sequencing reads. Gene annotation was conducted with the JGI pipeline, which identified 532,854 genes from the metagenomics assembly. The genes were functionally categorized using the Pfam, KEGG, and COG database.

**Figure 3:**
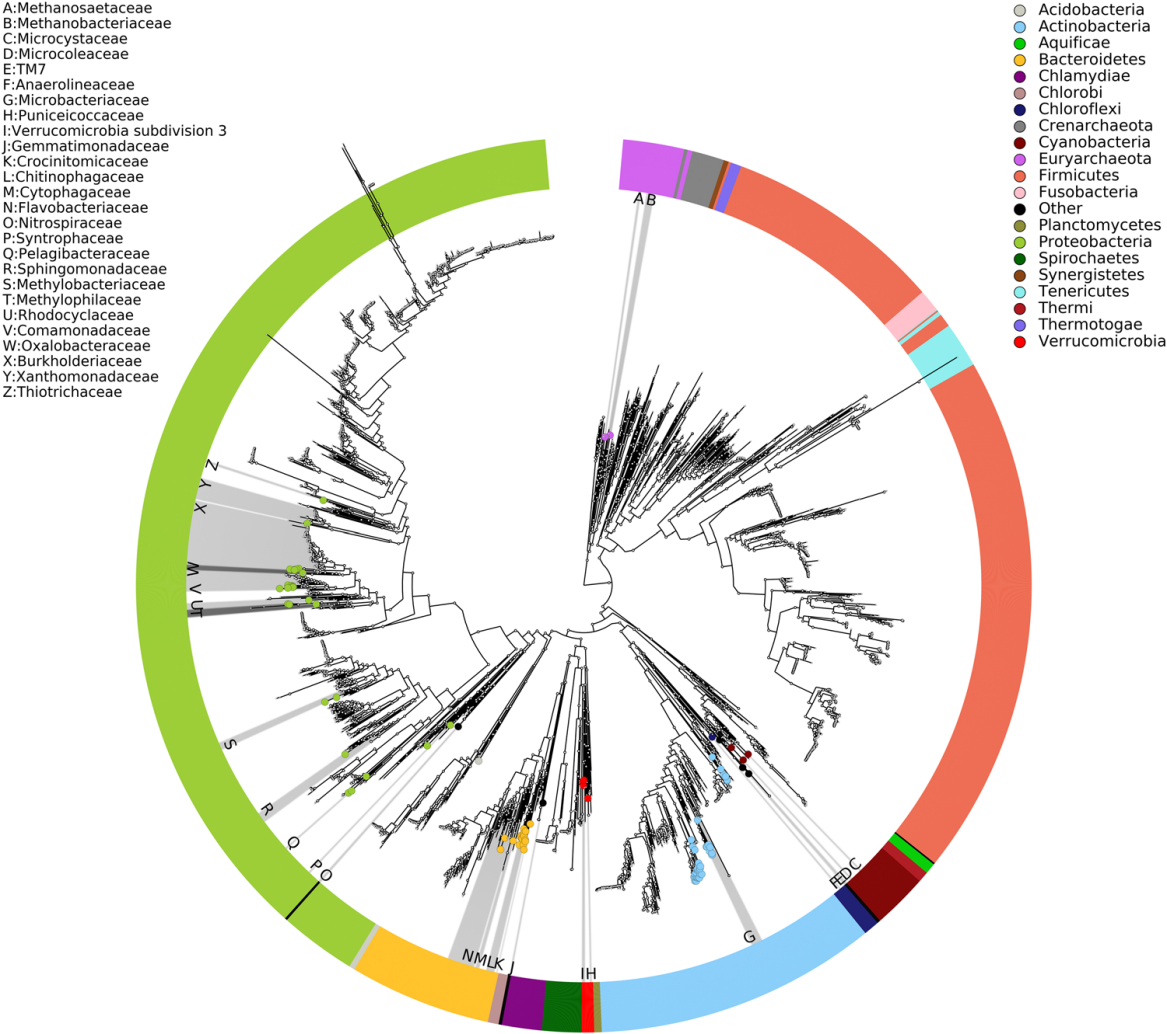
Phylogenetic assignment of assembled genome bins. The phylogenetic tree was obtained with PhyloPhlAn using 400 broadly conserved proteins to extract phylogenetic signal. Organisms are colored based on phyla.

Assembled contigs were organized into 291 GBs based on tetranucleotide sequence composition and different coverage patterns. After removing GBs with less than 50% completeness or over 20% contamination, phylogenetic analyses were conducted for the 95 GBs. The phylogenetic tree suggested that these 95 GBs belonged to 27 families from 11 phyla (Fig. 3, Supplementary Table S3). A total of 18 GBs were assigned to the genus level and 42 GBs were classified to the family level, while the remaining 35 GBs were classified to the phylum level due to the limitation of available related reference genomes.

### Antibiotic-resistant genes (ARGs) identification and antibiotic-resistant ontology (ARO) analysis

**Figure 4:**
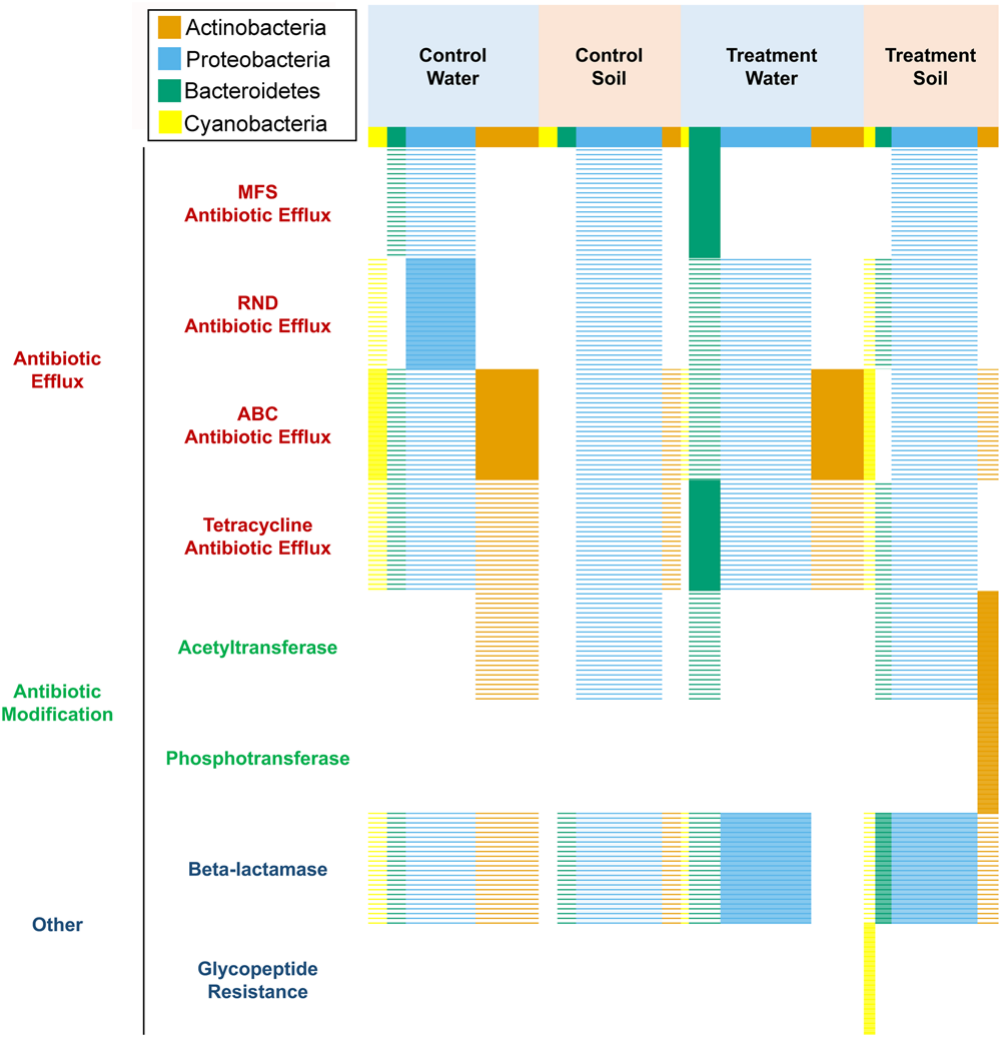
Binary heatmaps of major antibiotic resistance mechanisms organized by phylogeny within habitat. Sections of the heatmaps are colored once a particular AR mechanism is significantly enriched in a phylum (P value < 0.05, Fisher’s exact test).

The core Resfams database was used to identify ARGs from all samples collected, and a total of 603 AR genes were identified in the combined assembly. The identified ARGs were then assigned AROs and classified into broad functions, including major facilitator superfamily (MFS) antibiotic efflux, resistance-nodulation-cell division (RND) antibiotic efflux, ATP-binding cassette (ABC) antibiotic efflux, tetracycline antibiotic efflux, acetyltransferase, phosphotransferase, β-lactamase, and glycopeptide resistance. The Fisher’s exact test was used to identify enriched AR mechanism by bacterial phyla within habitat with P values < 0.05. As shown in Fig. 4, MFS and tetracycline antibiotic efflux were significantly enriched in Bacteroidetes from water collected from the treated system; ABC antibiotic efflux was enriched in Actinobacteria from water samples collected from the control and treated systems; and β-lactamase was enriched in Proteobacteria from treated ponds (both in sediment and in water). In addition, glycopeptide resistance was enriched only in Cyanobacteria present in sediment samples collected from the treated system.

### Genetic heterogeneity and selective sweep in microbial populations

**Figure 5:**
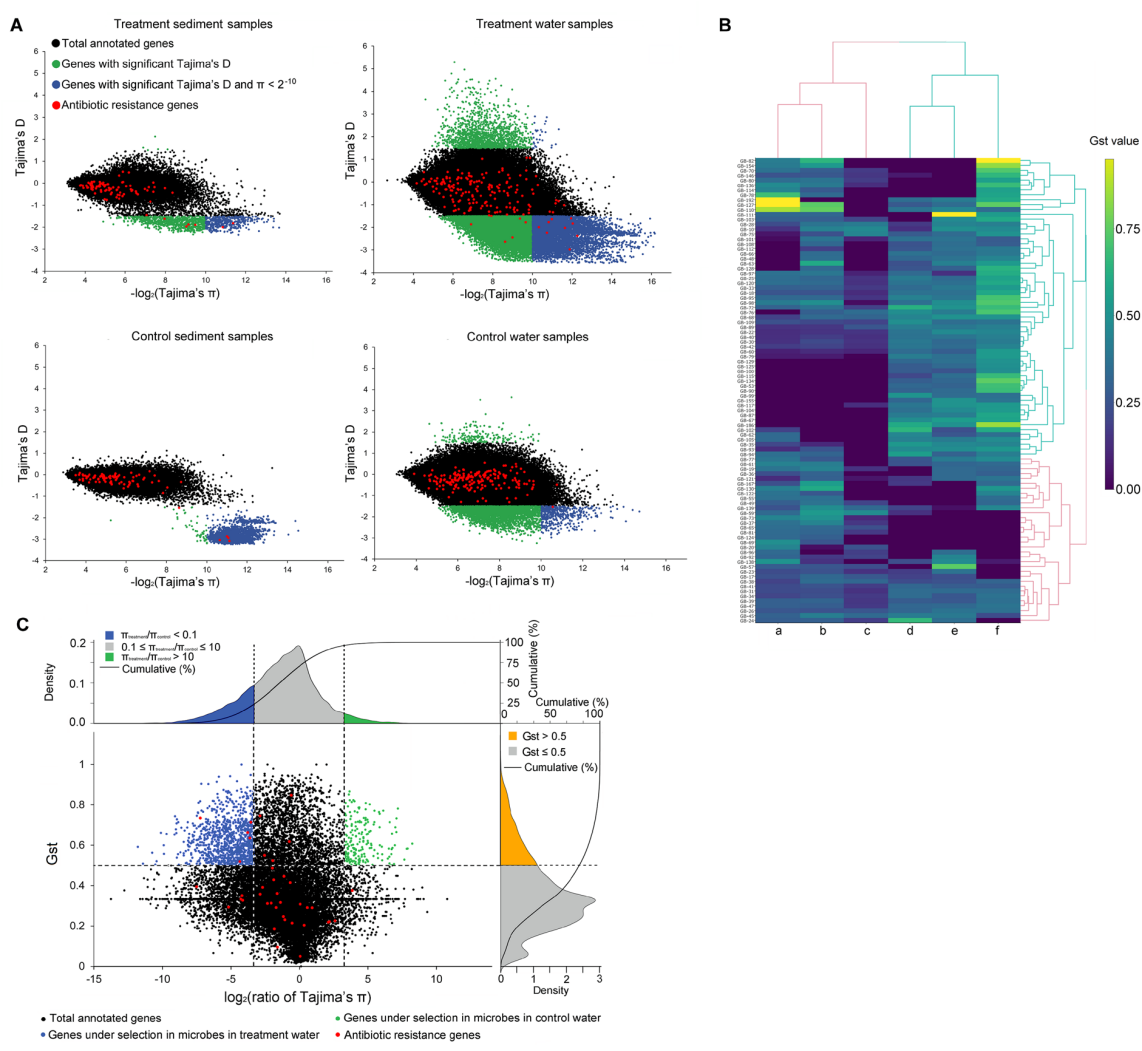
The analysis of genes under selection. (**A**) Gene-wise selective analysis by Tajima’s D test of the four populations. (**B**) GB-wise Gst analysis. a, control sediment against treated sediment; b, control water against treated sediment; c, control water against control sediment; d, treated water against treated sediment; e, treated water against control sediment; f, treated water against control water. (**C**) Gene-wise Gst analysis between microbial populations from treated water and control water samples.

The genome-wide scan of Tajima’s D was performed to screen for genes under directional evolution in the microbial populations present in all samples. Gene-specific selective sweeps were identified with Tajima’s test of neutrality and nucleotide diversity. Non-neutral genic loci were identified when π < 2^−10^ and Tajima’s D was greater than 1.5 or less than −1.5. As shown in Fig. 5A, a total of 452 (control water), 6,173 (treatment water), 2,828 (control sediment), and 385 (treatment sediment) genes were under positive selection (blue dots in Fig. 5A). A significantly higher number of genes (6,173) from the treatment water samples were identified as being under positive selection, suggesting that the micro-niche-adapted populations in treatment water were likely driven by stronger environmental selection. When taking a look at the distribution of AR genes in Fig. 5A, AR genes from treatment water spread over a wider range compared with that from the other three habitats. Two (treatment sediment), nine (treatment water), four (control sediment), and one (control water) AR genes were identified to be under positive selection.

To investigate the difference in genetic structure among microbial populations, all GBs were hierarchically clustered based on the Gst values calculated between every two types of samples (Fig. 5B).The Gst values calculated between the treatment water and the other three samples (column d, e, and f in Fig. 5B) clustered together and were greater than 0.25 in most of the GBs, suggesting that microbial populations in treatment water exhibited greater divergence and trended toward the fixation of alternative alleles compared to other samples.

Gene-level Gst values were also calculated to interpret genic selection occurring in the microbial populations present in the treated water samples. As shown in Fig. 5C, the total identified genes were divided into three groups based on gene-wise Gst values and levels of nucleotide diversity (Tajima’s π). A total of 1,046 genes had Gst values greater than 0.5 and π_treatment_/π_control_ less than 0.1 (blue dots in Fig. 5C); these genes were identified to be genes under selection in microbial populations present in treatment water samples. In contrast, only 180 genes were identified to be under selection in control water samples by showing Gst values greater than 0.5 and π_treatment_/π_control_ greater than 10 (green dots in Fig. 5C). The remainder of the genes were defined as having no obvious differences in allele frequency between treatment and control water samples (black dots in Fig. 5C). When taking a look at the AR genes (red dots in Fig. 5C), five AR genes were identified to be under selection in treatment water samples, and no AR gene was identified to be under selection from the control water samples.

### Functional analysis of genes under selection

Genes under selection in response to four environmental variables (treatment sediment, treatment water, control sediment, and control water) were collected for COG enrichment analysis and KEGG pathway analysis. As shown in Table 1, genes under selection in the treatment water sample mainly belonged to intracellular compartments and trafficking pathways (COG classes U and M), cell motility (class N), nucleotide transport and metabolism (class F), and defense mechanisms (class V). According to the KEGG database (Supplementary Table S4), many of these genes interact as parts of functional modules or pathways. A total of 23 functional modules/pathways related to environmental information processing, energy metabolism, and nucleotide metabolism were obtained at a high confidence cutoff (KEGG module completion ratio ≥ 50% and Q value < 0.05). When taking a look at the genes under selection identified from the control water samples, no significant pathway was detected from those genes.

**Table 1:** COG enrichment analysis of genes under positive selection.

COG classes	Population count^a^	Study count^b^	FDR
Genes under selection in treatment water identified by Gst			
(N) Cell motility	109	22	4.81E-05
(U) Intracellular trafficking, secretion, and vesicular transport	288	33	5.34E-03
(V) Defense mechanisms	151	29	7.56E-06
Genes under selection in control water identified by Tajima’s test of neutrality			
(L) Replication, recombination and repair	5,752	31	1.18E-04
Genes under selection in treatment water identified by Tajima’s test of neutrality			
(F) Nucleotide transport and metabolism	3,482	153	4.36E-04
(L) Replication, recombination and repair	6,411	288	8.30E-08
(M) Cell wall/membrane/envelope biogenesis	6,661	294	1.41E-07
(O) Post-translational modification, protein turnover, chaperones	4,712	195	8.09E-04
Genes under selection in control sediment identified by Tajima’s test of neutrality			
(M) Cell wall/membrane/envelope biogenesis	1,926	193	2.47E-07
(N) Cell motility	621	82	2.47E-07
(S) Function unknown	2,826	223	2.04E-02
(T) Signal transduction mechanisms	1,897	206	5.41E-10
(U) Intracellular trafficking, secretion, and vesicular transport	944	91	2.80E-03

^a^number of COG function term associated genes in the population gene set.

^b^number of COG function term associated genes in the selective swept gene set.

For the control sediment samples, as shown in Table 1, selective sweep genes in the control sediment sample were enriched in signal transduction (class T), cell motility and cell wall/membrane/envelope biogenesis (classes N and M) and intracellular trafficking (class U), as well as in some unknown functions (class S). Evaluation of the metabolic and physiological potentials revealed 70 KEGG modules/pathways that belong to broader categories such as carbohydrate and lipid metabolism, energy metabolism, and genetic information processing (Supplementary Table S4).

### Antibiotic-resistance genes under selection

The analysis of population differentiation and selective sweep revealed that 21 antibiotic-resistance genes were under selection in the four microbial populations. As shown in Table 2, 14 of the 21 antibiotic-resistance genes were identified in the treatment water samples. Most of these ARGs were antibiotic efflux transporters, including six ATP-binding cassette (ABC) antibiotic efflux pump genes, four RND antibiotic efflux pump genes, and one tetracycline resistance MFS efflux pump gene that can selectively pump out tetracycline or tetracycline derivatives. In contrast, only seven ARGs were identified to be under selection in the other three samples, which were also mainly characterized as antibiotic efflux pumps.

**Table 2:** AR genes under selection in the four samples.

Sample	GB	Gene name	Resfam Family	Tajima’s D	Gst	Tajima’s π
Treated water	GB-104	ABC transporter ATP-binding protein	ABC_efflux	−0.05	0.71	1.32E-02
	GB-18	ABC transporter ATP-binding protein	ABC_efflux	−0.11	0.52	4.36E-03
	GB-18	Multidrug transporter AcrB	RND_efflux	−0.32	0.64	4.41E-03
	GB-18	ABC transporter ATP-binding protein	ABC_efflux	−0.31	0.73	3.35E-04
	GB-186	ATP-binding protein MsbA	ABC_efflux	−2.98	—	2.67E-04
	GB-30	Antibiotic ABC transporter ATP-binding protein	ABC_efflux	−2.02	—	3.15E-04
	GB-33	Multidrug efflux pump	RND_efflux	0.21	0.66	2.27E-03
	GB-35	ABC transporter ATP-binding protein	ABC_efflux	−1.83	—	2.38E-04
	GB-35	Hydrophobe/amphiphile efflux-1 family RND transporter	RND_efflux	−1.64	—	4.97E-04
	GB-48	Hydrophobe/amphiphile efflux-1 family RND transporter	RND_efflux	−1.99	—	7.85E-04
	GB-48	Tetracycline resistance MFS efflux pump	tet_MFS_efflux	−1.61	—	2.53E-04
	GB-60	Efflux RND transporter periplasmic adaptor subunit	MexH	−2.24	—	5.88E-04
	GB-60	Multidrug efflux protein	MexW-MexI	−2.37	—	2.04E-04
	—	beta-lactamase	NDM-CcrA	−1.77	—	5.54E-04
Treated sediment	GB-17	Lipid A export permease/ATP-binding protein MsbA	msbA	−1.99	—	5.38E-04
	GB-17	ABC transporter ATP-binding protein	ABC_efflux	−1.82	0.26	3.78E-04
Control sediment	GB-20	MexH family multidrug efflux RND transporter periplasmic adaptor subunit	MexH	−2.87	—	4.87E-04
	GB-20	Multidrug transporter AcrB	RND_efflux	−2.93	—	4.66E-04
	GB-20	ABC transporter ATP-binding protein	ABC_efflux	−3.04	—	6.25E-04
	GB-20	Lipid A export permease/ATP-binding protein MsbA	msbA	−3.08	—	4.50E-04
Control water	—	MBL fold metallo-hydrolase	ClassB	−1.53	—	6.62E-04

The ARGs under selection in treated water samples belonged to eight GBs from six families belonging to the phyla of Actinobacteria, Bacteroidetes, Proteobacteria, and Verrucomicrobia. In contrast, ARGs identified from other samples were exclusively from a single GB. In treated sediment samples, the two ARGs under selection were from GB-17, genus Pedosphaera. In control sediment samples, selective sweep ARGs were exclusively present in GB-20, genus Beggiatoa.

### Gain and loss of antibiotic-resistance genes

As shown in Table 3, a total of 207 gene gains and losses were observed when comparing AR genes identified from treatment water with the AR genes identified from control water samples; while a total of 167 gene gains and losses were observed when comparing the AR genes identified from the treatment sediment with the control sediment samples. A total of 262 unique AR gene gains and losses were identified in water and sediment samples collected from the treatment system when compared with their corresponding control samples.

**Table 3:** Copy number changes of the AR genes identified in the treatment water and sediment samples collected from the in-pond raceways production system.

Resfams family	Treatment water			Treatment sediment		
	Gene gain	Gene loss	Sub-total	Gene gain	Gene loss	Sub-total
ABC_efflux	38	58	96	36	41	77
RND_efflux	16	12	28	12	15	27
ClassB	10	13	23	12	7	19
tet_MFS_efflux	8	9	17	8	3	11
ClassA	4	7	11	6	3	9
msbA	6	4	10	1	4	5
MexH	3	6	9	3	4	7
MexE	1	2	3	0	1	1
ClassD	2	0	2	0	1	1
MexW-MexI	0	2	2	1	2	3
NDM-CcrA	1	0	1	0	1	1
ArmA_Rmt	0	1	1	1	0	1
MexX	0	1	1	1	0	1
vanR	1	0	1	1	0	1
AAC3	0	1	1	1	1	2
macB	0	1	1	1	0	1
Total	90	117	207	84	83	167

**Figure 6:**
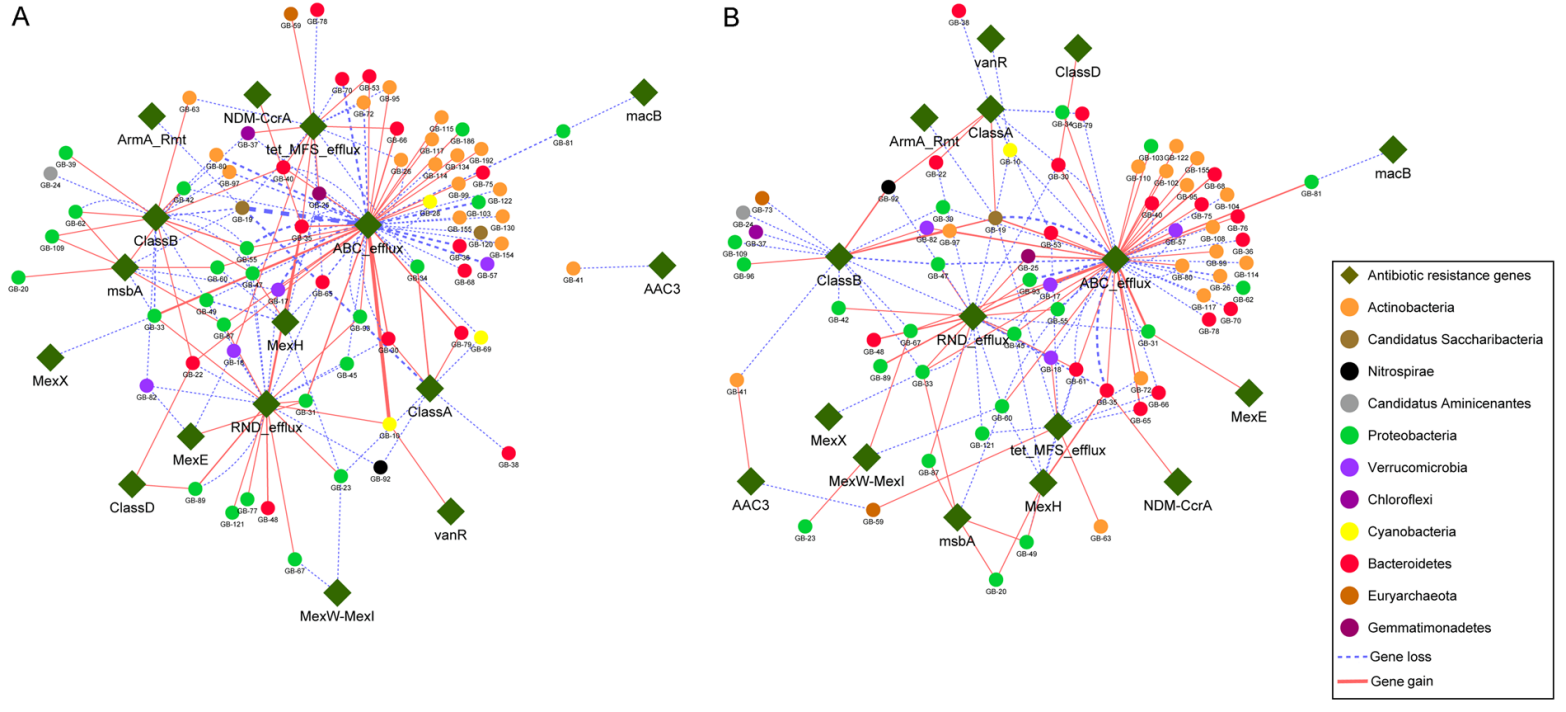
AR gene gains and losses in (**A**) water samples and (**B**) sediment samples. The blue lines represent gene loss when comparing treatment samples with control samples, while the red lines represent gene gains when comparing treatment samples with control samples. The width of each connection line between the AR genes and the phylum is proportional to the number of genes with changed frequency. The wider the line, the more gene gains or losses identified in that GB.

For the treatment water sample, frequencies of over 200 ARGs were changed, including 90 gains and 117 losses. Almost half of these AR genes belong to the class of ABC antibiotic efflux pumps, including 38 gains and 58 losses. In addition to ABC efflux pumps, the other major AR gene classes include resistance-nodulation-cell division (RND) efflux pumps, tetracycline resistance major facilitator superfamily (MFS) efflux pumps, and AR genes against β-lactams. Phylum-specific patterns of AR gene gains and losses were also observed in treatment water samples (Fig. 6). For example, gene gains and losses of ABC efflux pumps were observed mainly in the phyla of Actinobacteria and Bacteroidetes. Copy number changes of class B β-lactamases were mainly observed in the phylum of Proteobacteria. The gain and loss of tetracycline resistance MFS efflux pumps were mostly identified in the phylum of Bacteroidetes.

For treatment sediment samples, a total of 167 AR genes with 84 gains and 83 losses were identified from 67 GBs belonging to 11 phyla. The pattern of gene gains and losses and their correlations with different phyla were similar to that of the treatment water samples, with most of the AR genes characterized as ABC efflux pumps, RND efflux pumps, and tetracycline MFS efflux pumps. As noted in Fig. 6, efflux pumps were the dominant AR functions that had significant gains or losses when comparing the treatment water or sediment samples with their corresponding control samples.

## Discussion

Aquaculture has been a fast-growing industry as the demand for fish and seafood dramatically increases throughout the world. As a result, traditional pond farming systems in many regions are now slowly being replaced by modern high-intensity systems^28^. However, growing concerns are expressed about the potential effects of ever-increasing aquaculture waste on the microbial ecosystenms in these systems. Uneaten feed, excreta, and chemicals are all processed biochemically in the aquaculture production system, alterating the physical and chemical properties of the environment. Chopin *et al*. estimated that 49.3 kg N and 7.0 kg P per ton of fish are released into the water column per year^29^. In addition, the extensive use of antibiotics in aquaculture induces the emergence and dissemination of diverse AR genes in microorganisms, which has already undermined the treatment efficiency of these antibiotics. According to the World Health Organization, approximately half of the world’s antibiotics are used in agricultural animals^30,31^, leading to increasing antibiotic-resistance risks in aquaculture production ecosystems. Without proper management, aquaculture pollution accidents caused by aquaculture wastes, such as the uneaten feed and chemical and antibiotic treatments, may cause economic losses totaling millions of dollars^32^. In recent years, the monitoring and manipulating of microbial communities in aquaculture environments have shown great potential. However, additional information about the microbial populations present in complex aquacultural production systems and their genome-wide changes is still missing. There is an urgent need to understand the adaptations that drive the associations of bacteria to particular ecological niches in aquaculture environments, as well as genetic factors underlying the heterogeneity in antimicrobial resistance.

In the United States, catfish production plays an important role in the aquaculture industry, constituting 68% of total domestic freshwater production in 2015^33^. IPRS catfish production systems have become more and more popular in the United States because of their low product cost and high production yield. Such system serves as a great model for studying the dynamic genetic repertoire of microbial communities and the process of environmental selection in metapopulations. In this study, water and sediment samples from both the catfish IPRS high-intensity production systems (treatment) and the control watershed were collected for metagenomic sequencing. The taxonomic analyses revealed that the aquatic microbial population has a lower level of species diversity compared to the sediment samples, regardless of whether the water is from the treatment system or the control watershed. To examine the extent of taxonomic abundance changes between the treatment and control bacterial communities, water and sediment samples were compared separately. In the IPRS production systems, the bacterial communities found in water samples undergo more dramatic changes than the bacterial communities found in sediment (Fig. 2). The bacteria enriched in aquacultural water exhibited much lower species diversity than the bacteria in control water, indicating the simplification of microbial populations caused by the aquaculture. The significantly higher concentrations of the major photosynthetic clades, such as Cyanobacteria, Chlorobi, and Chloroflexi, suggest that the eutrophication of aquacultural water may result in the reduction of autotrophic bacteria and the reduction of the microbial ecosystem’s functional diversity. Similar results have been observed in other studies that evaluated the effects of aquaculture activities on microbial assemblages^34,35^. It has been found by previous studies that the organic load generated from aquaculture production activities significantly reduced the bacterial diversity and altered the assemblage composition^36,37^.

In this study, bacterial genomes were reconstructed from a combined assembly of all the samples. Contigs from the combined assembly were organized and curated into 95 GBs. Closely related genomes were distinguished based on unique read coverage patterns throughout the collected samples^38^. Taxonomic assignment showed that none of the GBs could be assigned to species level, only 18 GBs were assigned to the genus level, while 35 GBs were assigned to the phylum level, revealing that most of the microbial species in the community were not previously characterized at the genomic level.

According to Bendall *et al*.’s study, reads mapped to each GB are regarded as sequence-discrete populations that can be used for genetic variation and selective sweep analysis^39^. The Gst, Tajima’s D, and π tests were used to identify potential genetic loci affected by selection. Both the Gst and Tajima’s indices revealed that genomic regions appeared to sweep independently without purging diversity of the rest of the genome. Controversy exists as to whether genome-wide sweeps or gene-specific sweeps are driven by selection. Evidence of genome-wide sweep has been reported in Bendall *et al*.’s study, where nearly all of the genes from a green sulfur bacteria swept slowly over several years^39^. In contrast, gene-specific sweep has been supported by studies on multiple microbial populations^40–42^. As reviewed by Shapiro *et al*., gene-specific sweep may be more common when recombination is frequent and the selection is moderate^43^. A study on negative frequency-dependent selection (NFDS) revealed that gene-specific selective sweeps are expected to be a major and general phenomenon in prokaryotes due to potential NFDS caused by ubiquitous viruses. In this study, the largest number of selective swept genes was identified in water samples from IPRS, suggesting that obvious divergence and fixation of alternative alleles were caused by aquaculture production actives.

Genes under selection in different samples were associated with specific functions such as protein biogenesis, cell motility, nucleotide metabolism, and defense mechanisms, leading to the hypothesis that such genes are heavily involved in the microbial adaptation in the IPRS environment. In the IPRS water sample, the multidrug efflux pump pathway was significantly enriched, suggesting that a recent selective sweep caused by the addition of oxytetracycline removed most of the variations in the region. In addition to SNP dynamics, patterns of gene gain and loss within microbial populations were revealed by gene frequency changes. The relative abundance of over 260 AR genes was changed in the treatment sediment and water samples. Despite a small number of AR genes that belong to metallo-beta-lactamase (MBL) fold metallo-hydrolase, most of them are related to antibiotic efflux pumps. Previous studies have revealed that the exposure of bacteria to antibiotics may induce SOS responses to disseminate AR genes^44–46^. Thus, the observed AR gene gains and losses are likely associated with horizontal gene transfer or integron recombination. This phenomenon may also be explained by the balancing selection hypothesis^47^. The general tempo of gene transfer is affected by two opposing forces: the benefit accrued through acquisition of foreign genes and the potential deleterious effects of invasion by detrimental genetic elements. The antibiotics distributed in the environment may have altered the balance of the two forces. Therefore, bacteria lineage with a greater porosity to gene transfer have an inherent advantage in the expression of resistance genes^48,49^.

In summary, this study examined the ecological and evolutionary patterns of the bacterial communities present in an IPRS high-intensity catfish production system by conducting metagenomic sequencing. By comparing microbial compositions and genetic structures, we observed substantial genetic heterogeneity within genetically and ecologically distinguishable populations. Population genetic summary statistics calculated for the collected samples suggested that gene-specific sweep is more common than genome-wide sweep in microbial populations present in aquaculture production system. Over 6,000 genes were identified under selective pressure in the IPRS water samples, suggesting that aquaculture wastes, especially therapeutic antibiotics, generate significant impacts on microbial population genetic structures. Furthermore, over 200 AR gene gains and losses were identified by analyzing the changes of gene frequencies, with most of the AR genes characterized as ABC efflux pumps, RND efflux pumps, and tetracycline MFS efflux pumps. This discovery highlighted the importance of better understanding the short-term and long-term impacts generated by the therapeutic treatments used in aquaculture. This work is the first to provide an in-depth evaluation of the genetic adaptive patterns of aquatic microbial communities present in high-intensity catfish production systems. The information generated will directly benefit future works on determining genetic factors underlying the heterogeneity in antimicrobial resistance and on the development of intervention strategies for mitigating antibiotic resistance risks.

## Methods

### The high-intensive production system and sample collection

The IPRS high-intensity catfish culture system selected for this study was stocked with channel catfish (*Ictalurus punctatus*) at a rate of ~25,000 fish/ha. The water in this high-intensity system was taken from a watershed reservoir, which was also sampled and used as the “control.” Fish in this high-intensity production system were infected with columnaris disease and treated with Terramycin orally through medicated feed at 50 mg/kg of fish per day for 10 days. Daily care, pond operations and management, application of medicated feed as well as the sample collections were performed following the procedures outlined in SOP 2015–2705, a standard operation procedure manual approved by the Institutional Animal Care and Use Committee (IACUC) at Auburn University. Four 1-L water and four 50-gram sediment samples were collected from the treated production system two months after the addition of Terramycin (“treatment” samples). Four water samples and four sediment samples were collected in the same time from the control watershed (“control” samples).

### DNA extraction, library preparation and sequencing

For every water sample, 1-L of collected water was first filtered through 0.2-um filters (EMD Millipore, Temecula, CA). The filtered membranes were then cut with scissors into small pieces for DNA extraction using a PowerSoil® DNA isolation kit (MoBio Laboratories, Carlsbad, CA) according to the manufacturer’s instructions. The DNA of each sediment sample was extracted using the same kit. Equal amounts of DNA (~1000 ng/sample) extracted from every water sample collected from either the treated system or the control system were pooled for sequencing. Equal amounts of DNA (~1000 ng/sample) extracted from every sediment sample collected from the treated or the control system, were pooled for sequencing.

The library construction and sequencing were conducted at the HudsonAlpha Genomic Services Lab (Huntsville, AL). Genomic libraries were prepared using the Paired-end Sequencing Sample Preparation Kit (Illumina, San Diego, CA) according to the manufacturer’s instructions. The four prepared DNA libraries (treatment sediment, treatment water, control sediments, control water) were sequenced on one lane of the Illumina HiSeq. 2000 platform to generate 100-bp paired-end reads.

### Taxonomy classification and differential abundance analysis

Raw reads were first evaluated for quality using FastQC (version 0.11.5)^50^. Reads were then trimmed using Trimmomatic (version 0.36)^51^. Ambiguous nucleotides (N’s), extreme short reads (<25 nt), and low-quality bases were trimmed with a sliding window method, bases within a window size of 4 were cut once the average quality was less than 15 in the Phred scale. The filtered reads were subjected to taxonomy classification by Kaiju (version 1.4)^52^ with the representative set of bacterial and archaeal genomes from the proGenomes database^53^. The phylogenetic analyses of the microbial composition were conducted by using Phylosift (version 1.0.1)^54^. Sample distances and Shannon diversity index were estimated using MetagenomeSeq (version 1.6)^55^. Genera with significantly different abundance between the “treatment” and “control” samples were identified using edgeR (version 3.16.5) with FDR corrected P values < 0.05^56^.

### De novo metagenome assembly and gene annotation

Filtered reads from all samples were pooled together and assembled using Megahit (version 1.1)^57^ with k-mer size from 27 to 99 with a step of 10. Contigs with a length over 2.5 kbp were organized into genome bins (GBs) using MetaBat (version 0.32.4)^58^ based on the tetranucleotide sequence composition and overall contig coverage patterns obtained from backtrack alignment files. Reads from each sample were mapped to the assembly using the Burrows-Wheeler aligner (BWA) employing the backtrack alignment algorithm with a minimum of 95% sequence identity^59^. To assess the completeness of GBs and to reduce the incorrectly binning contigs from different organisms, overlapping sets of ubiquitous and single-copy genes within a phylogenetic lineage were estimated in each GB by CheckM (version 1.0.6)^60^. GBs with less than 50% completeness or over 20% contamination were excluded from phylogenetic analysis.

Gene prediction and annotation of metagenomic reconstructions were performed using the DOE Joint Genome Institute’s Integrated Microbial Genome database tool (version 4.15.1)^61^. Briefly, open read frames were predicted by Prodigal (version 2.6.3)^62^ and Prokaryotic GeneMark.hmm (version 2.8)^63^. Conserved protein families and domains were identified using BLASTP search against COG^64^, Pfam^65^, and KEGG Orthology (KO) database^66^ with the cutoff of e value < 1e-10. KEGG pathway modules were identified using MAPLE (version 2.3.0)^67^. Antibiotic resistance genes (ARGs) were identified using the Resfams database (version 1.2)^68^.

### Phylogenetic analysis and taxonomic analysis of metagenomic assemblies

The taxonomic identities of identified GBs and their evolutionary relationships with 3,171 known microbial genomes were determined using PhyloPhlAn pipeline (version 0.99)^69^. GBs were assigned to the finest taxonomic level for which all marker genes agreed, ranging from the phylum level for some genomes to the genus level for others. Predicted genes within each GB were also checked by sequence similarity to the non-redundant (NR) database using BLASTN. The taxonomic assignment of the best match generated by BLASTN was retrieved for validation of the results generated from PhyloPhlAn.

### Single-nucleotide polymorphism (SNP) identification and analysis

SNPs were identified by mapping reads with over 95% identity from each sample to reference genomes using BWA. SNPs were identified with a minimal coverage of 5, minimal average base quality of 30, and minor allele frequency of 0.05. To ensure that only the high-confidence SNPs were examined, SNPs were removed once its coverage is over 1.5 interquartile ranges below the first quartile of above the third quartile. Allele frequencies were calculated as the number of reads that carry the reference or alternate alleles.

Tajima’s π and D were calculated to evaluate the nucleotide diversity and identify mutations under selection. Non-neutral genic loci (the ones under selection or experienced gene-specific sweeps) were identified when π < 2^−10^ and Tajima’s D was over 1.5 or less than −1.5. To measure the genetic structure changes between microbial populations, the Fixation index (Gst) was calculated by comparing the allele frequencies of each GB associated with every two sample types. Gst is a measurement of population genetic differentiation^70^, it measures the differentiation fairly well especially when heterozygosity is low^71^. In this study, genes with Gst > 0.5 and π_treatment_/π_control_ < 0.1 were identified as genes with spread of adaptive alleles and recent purge of diversity in treatment water samples; genes with Gst > 0.5 and π_treatment_/π_control_ > 10 were identified as genes under selection in control water samples; the remainder of the genes were defined as having no obvious differences in allele frequency between treatment and control water samples.

### Gene gain and loss

To identify genes with significantly changed abundance between the treatment and control samples, gene coverage and frequency were calculated for each GB of every sample. Gene coverage was determined by normalizing the number of mapped metagenomics reads by the gene length. Gene frequency was determined for each gene by dividing its coverage by the median coverage of all genes within a GB, which implies the copy number of every gene present in each cell within one microbial population^39^. Genes were considered as gained or lost once their frequencies changed by magnitudes greater than 0.4 between the treatment and control samples.

### Data availability

All the sequencing data were deposited at the NCBI BioProject with accession PRJNA394151.

## Electronic supplementary material

Supplementary information
