# Supplementary material for: Genetic adaptation of microbial populations present in high-intensity catfish production systems with therapeutic oxytetracycline treatment
Source: Sci Rep. 2017 Dec 13;7:17491. doi: 10.1038/s41598-017-17640-3 (PMC5727513; doi:10.1038/s41598-017-17640-3)
Supplement: Supplementary file 1 — Supplementary information [file 41598_2017_17640_MOESM1_ESM.pdf]

Supplementary information

Genetic adaptation of microbial populations present in high-intensity catfish production systems with therapeutic oxytetracycline treatment

Qifan Zeng[1], Xiangli Tian[2], and Luxin Wang[1*]

[1]Food Microbiology and Safety Lab, Department of Animal Sciences, Auburn University, Auburn, Alabama, USA

[2]The Key Laboratory of Mariculture, Ocean University of China, Qingdao, China

[*]Correspondence: E-mail: lzw0022@auburn.edu

Supplementary Table S1. Summary of the sequencing data before and after trimming.

Pond	Type	Number of raw reads (million)	Read length (nt)	Number of reads after trim (million)	Average length after trim (nt)
IPRS Treatment	Sediment	62.37	100	60.81	95.98
	Water	74.44	100	72.85	95.96
Control	Sediment	88.11	100	85.01	95.89
	Water	53.58	100	51.50	95.83
Total	-	278.50	-	270.17	-

Supplementary Table S2. Statistics of differentially abundant genera (FDR P values < 0.05) between sediment and water samples collected from the treated system and the control water reservoir.

Sample type	Bacteria and Archaea		Virus	
	Greater abundance in treatment	Greater abundance in control	Greater abundance in treatment	Greater abundance in control
Water samples	149	190	72	26
Sediment samples	6	4	7	1

Supplementary Table S3. Genome bins reconstructed from metagenomic-combined assembly.

Genome bin ID	Genome bin family name	Genome bin size (Mb)	Genome bin completeness (%)	Genome bin contamination (%)
GB-192	unclassified Actinobacteria	0.48	53.45	4.74
GB-186	Burkholderiaceae	0.51	50	0
GB-167	Pelagibacteraceae	0.61	75.5	1.31
GB-155	unclassified Actinobacteria	0.75	62.57	1.72
GB-154	unclassified Actinobacteria	0.76	57.7	2.63

GB-146	TM7	0.82	53.22	1.16
GB-139	unclassified Actinobacteria	0.9	56.83	2.11
GB-138	unclassified Actinobacteria	0.91	63.88	1.05
GB-136	unclassified Actinobacteria	0.93	66.54	2.59
GB-134	unclassified Actinobacteria	0.95	72.84	0
GB-130	unclassified Actinobacteria	0.99	67.75	3.24
GB-129	unclassified Actinobacteria	1	69.27	8.99
GB-128	Microbacteriaceae	1.01	68.42	7.02
GB-127	unclassified Actinobacteria	1.03	68	1.87
GB-125	Microbacteriaceae	1.06	78.74	2.73
GB-124	unclassified Actinobacteria	1.06	75.49	4.38
GB-122	Microbacteriaceae	1.09	78.22	0
GB-121	Pelagibacteraceae	1.09	84.14	6.74
GB-120	TM7	1.1	87.5	0
GB-117	unclassified Actinobacteria	1.13	65.58	2.11
GB-115	unclassified Actinobacteria	1.15	81.7	1.14
GB-114	unclassified Actinobacteria	1.18	79.31	1.72
GB-112	Microbacteriaceae	1.22	76.21	1.34
GB-111	Methylophilaceae	1.23	90.54	7.37
GB-110	unclassified Actinobacteria	1.23	85.24	2.02
GB-109	Methylophilaceae	1.29	80.99	4.08
GB-108	unclassified Actinobacteria	1.29	71.13	7.18
GB-105	Microbacteriaceae	1.34	90.11	2.05
GB-104	Microbacteriaceae	1.35	87.49	1.85
GB-103	Methylobacteriaceae	1.36	56.04	1.39
GB-102	unclassified Actinobacteria	1.38	81.97	7.27
GB-101	unclassified Actinobacteria	1.4	79.65	2.59
GB-100	Microbacteriaceae	1.41	94.7	1.27
GB-99	unclassified Actinobacteria	1.41	74.28	8.43
GB-98	unclassified Actinobacteria	1.43	66.38	1.72
GB-97	unclassified Actinobacteria	1.43	67.33	2.66
GB-96	Syntrophaceae	1.44	68.45	2.26
GB-95	unclassified Actinobacteria	1.45	81.03	6.55
GB-94	unclassified Actinobacteria	1.47	80.43	18.41
GB-93	Burkholderiaceae	1.48	73.62	3.45
GB-92	Nitrospiraceae	1.53	59.13	13.68
GB-90	Microbacteriaceae	1.54	88.3	14.93
GB-89	Rhodocyclaceae	1.54	94.56	0.42
GB-87	Oxalobacteraceae	1.58	61.72	2.12
GB-82	Puniceicoccaceae	1.59	96.62	0.68
GB-81	unclassified Rickettsiales	1.61	95.45	3.57
GB-80	unclassified Actinobacteria	1.62	81.47	2.92
GB-79	Flavobacteriaceae	1.65	70.04	0.71

GB-78	unclassified Bacteroidetes	1.65	75.52	4.92
GB-77	Methylophilaceae	1.68	61.52	6.9
GB-76	Flavobacteriaceae	1.73	59.81	0
GB-75	unclassified Bacteroidetes	1.75	61.13	12.07
GB-73	Methanosaetaceae	1.79	82.84	2.61
GB-72	unclassified Actinobacteria	1.81	59.97	5.53
GB-70	unclassified Bacteroidetes	1.83	93.11	0.87
GB-69	Leptolyngbyaceae	1.84	68.86	1.3
GB-68	Chitinophagaceae	1.9	87.36	0.49
GB-67	Burkholderiaceae	1.9	94.26	7.48
GB-66	Chitinophagaceae	1.92	53.45	0.69
GB-65	Chitinophagaceae	1.93	95.07	0.27
GB-63	unclassified Actinobacteria	1.97	76.81	13.53
GB-62	Comamonadaceae	1.98	75.08	1.63
GB-61	Chitinophagaceae	1.99	73.94	19.8
GB-60	Burkholderiaceae	2.01	93.87	0
GB-59	Methanobacteriaceae	2.02	98.55	6.4
GB-57	Verrucomicrobia subdivision 3	2.04	85.95	3.1
GB-55	Comamonadaceae	2.08	90.2	5.91
GB-53	Chitinophagaceae	2.12	71.72	6.9
GB-49	Xanthomonadaceae	2.2	85.75	1.99
GB-48	Chitinophagaceae	2.22	91.59	0.63
GB-47	Comamonadaceae	2.22	82.6	15.19
GB-45	Comamonadaceae	2.33	79.95	7.34
GB-42	Comamonadaceae	2.46	98.16	0.3
GB-41	unclassified Actinobacteria	2.47	55.59	15.04
GB-40	Crocinitomicaceae	2.48	100	5.17
GB-39	Comamonadaceae	2.49	78.66	6.52
GB-38	Chitinophagaceae	2.51	64.4	0.82
GB-37	Anaerolineaceae	2.59	65.23	1.82
GB-36	Chitinophagaceae	2.6	80.28	6.3
GB-35	Cytophagaceae	2.63	94.94	0.61
GB-34	Sphingomonadaceae	2.65	98.59	2.95
GB-33	unclassified Burkholderiales	2.73	95.19	1.11
GB-31	Methylobacteriaceae	2.92	97.28	0.91
GB-30	Chitinophagaceae	3.03	99.51	0.25
GB-28	Microcystaceae	3.09	62.66	2.41
GB-26	unclassified Actinobacteria	3.15	61.95	6.41
GB-25	Gemmatimonadaceae	3.31	83.1	6.9
GB-24	unclassified Aminicenantes	3.46	93.42	5.56

GB-23	unclassified Deltaproteobacteria	3.49	81.98	18.42
GB-22	Chitinophagaceae	3.59	97.78	2.55
GB-20	Thiotrichaceae	3.7	93.21	2.11
GB-19	TM7	3.76	97.1	1.98
GB-18	Verrucomicrobia subdivision 3	3.86	96.28	4.19
GB-17	Verrucomicrobia subdivision 3	4.45	97.97	4.08
GB-10	Microcoleaceae	6.67	96.45	0.44

Supplementary Table S4. KEGG functional module analysis of genes under positive selection.

Function category	KEGG ID	Module name	MCR %[a]	Q-value[b]
Genes under selection in treatment water identified by Gst				
ABC-2 type and other transport systems	M00258	Putative ABC transport system	100	0
	M00254	ABC-2 type transport system	100	0
Central carbohydrate metabolism	M00307	Pyruvate oxidation, pyruvate => acetyl-CoA	100	0
	M00005	PRPP biosynthesis, ribose 5P => PRPP	100	0
Mineral and organic ion transport system	M00185	Sulfate transport system	100	0
Pyrimidine metabolism	M00052	Pyrimidine ribonucleotide biosynthesis, UMP => UDP/UTP,CDP/CTP	100	0
Genes under selection in treatment water identified by Tajima's test of neutrality				
ABC-2 type and other transport systems	M00250	Lipopolysaccharide transport system	100	0
	M00255	Lipoprotein-releasing system	100	0
	M00256	Cell division transport system	100	0
ATP synthesis	M00155	Cytochrome c oxidase, prokaryotes	100	0

Branched-chain amino acid metabolism	M00019	Valine/isoleucine biosynthesis, pyruvate => valine / 2-oxobutanoate => isoleucine	100	0
Carbon fixation	M00579	Phosphate acetyltransferase-acetate kinase pathway, acetyl-CoA => acetate	100	0
Central carbohydrate metabolism	M00010	Citrate cycle, first carbon oxidation, oxaloacetate => 2-oxoglutarate	100	0
	M00005	PRPP biosynthesis, ribose 5P => PRPP	100	0
Cysteine and methionine metabolism	M00021	Cysteine biosynthesis, serine => cysteine	100	0
Drug efflux transporter/pump	M00707	Multidrug resistance, MdlAB/SmdAB transporter	100	0
	M00647	Multidrug resistance, efflux pump AcrAB-TolC/SmeDEF	50	0.018
Fatty acid metabolism	M00082	Fatty acid biosynthesis, initiation	100	0
	M00086	beta-Oxidation, acyl-CoA synthesis	100	0
Other carbohydrate metabolism	M00793	dTDP-L-rhamnose biosynthesis	100	0
Phosphate and amino acid transport system	M00222	Phosphate transport system	100	0
RNA polymerase	M00183	RNA polymerase, bacteria	100	0
Serine and threonine metabolism	M00020	Serine biosynthesis, glycerate-3P => serine	100	0
Two-component regulatory system	M00434	PhoR-PhoB (phosphate starvation response) two-component regulatory system	100	0
Genes under selection in control sediment identified by Tajima's test of neutrality				
ABC-2 type and other transport systems	M00250	Lipopolysaccharide transport system	100	0
	M00320	Lipopolysaccharide export system	100	0
	M00252	Lipooligosaccharide transport system	100	0
	M00259	Heme transport system	100	0

	M00256	Cell division transport system	100	0
	M00258	Putative ABC transport system	100	0
	M00254	ABC-2 type transport system	100	0
Aminoacyl tRNA	M00360	Aminoacyl-tRNA biosynthesis, prokaryotes	100	0
Arginine and proline metabolism	M00015	Proline biosynthesis, glutamate => proline	100	0
Aromatic amino acid metabolism	M00022	Shikimate pathway, phosphoenolpyruvate + erythrose-4P => chorismate	100	0
ATP synthesis	M00149	Succinate dehydrogenase, prokaryotes	100	0
	M00151	Cytochrome bc1 complex respiratory unit	100	0
Bacterial secretion system	M00330	Adhesin protein transport system	100	0
	M00331	Type II general secretion pathway	100	0
	M00336	Twin-arginine translocation (Tat) system	100	0
Branched-chain amino acid metabolism	M00535	Isoleucine biosynthesis, pyruvate => 2-oxobutanoate	100	0
	M00432	Leucine biosynthesis, 2-oxoisovalerate => 2-oxoisocaproate	100	0
Carbon fixation	M00166	Reductive pentose phosphate cycle, ribulose-5P => glyceraldehyde-3P	100	0
	M00169	CAM (Crassulacean acid metabolism), light	100	0
	M00579	Phosphate acetyltransferase-acetate kinase pathway, acetyl-CoA => acetate	100	0
Central carbohydrate metabolism	M00001	Glycolysis (Embden-Meyerhof pathway), glucose => pyruvate	100	0
	M00002	Glycolysis, core module involving three-carbon compounds	100	0

	M00003	Gluconeogenesis, oxaloacetate => fructose-6P	100	0
	M00307	Pyruvate oxidation, pyruvate => acetyl-CoA	100	0
	M00009	Citrate cycle (TCA cycle, Krebs cycle)	87.5	0
	M00010	Citrate cycle, first carbon oxidation, oxaloacetate => 2-oxoglutarate	100	0
	M00011	Citrate cycle, second carbon oxidation, 2-oxoglutarate => oxaloacetate	80	0
	M00006	Pentose phosphate pathway, oxidative phase, glucose 6P => ribulose 5P	100	0
	M00005	PRPP biosynthesis, ribose 5P => PRPP	100	0
Cofactor and vitamin biosynthesis	M00125	Riboflavin biosynthesis, GTP => riboflavin/FMN/FAD	100	0
	M00115	NAD biosynthesis, aspartate => NAD	100	0
	M00120	Coenzyme A biosynthesis, pantothenate => CoA	100	0
	M00118	Glutathione biosynthesis, glutamate => glutathione	100	0
Cysteine and methionine metabolism	M00021	Cysteine biosynthesis, serine => cysteine	100	0
DNA polymerase	M00260	DNA polymerase III complex, bacteria	100	0
Drug resistance	M00742	Aminoglycoside resistance, protease FtsH	100	0
Fatty acid metabolism	M00082	Fatty acid biosynthesis, initiation	100	0
	M00083	Fatty acid biosynthesis, elongation	100	0
	M00086	beta-Oxidation, acyl-CoA synthesis	100	0

Lipid metabolism	M00093	Phosphatidylethanolamine (PE) biosynthesis, PA => PS => PE	100	0
Lipopolysaccharide metabolism	M00063	CMP-KDO biosynthesis	100	0
	M00064	ADP-L-glycero-D-manno-heptose biosynthesis	100	0
Lysine metabolism	M00016	Lysine biosynthesis, succinyl-DAP pathway, aspartate => lysine	100	0
Metallic cation, iron-siderophore and vitamin B12 transport system	M00240	Iron complex transport system	100	0
	M00242	Zinc transport system	100	0
	M00246	Nickel transport system	100	0
Mineral and organic ion transport system	M00185	Sulfate transport system	100	0
	M00189	Molybdate transport system	100	0
	M00299	Spermidine/putrescine transport system	100	0
	M00208	Glycine betaine/proline transport system	100	0
Other carbohydrate metabolism	M00012	Glyoxylate cycle	100	0
	M00549	Nucleotide sugar biosynthesis, glucose => UDP-glucose	100	0
	M00793	dTDP-L-rhamnose biosynthesis	100	0
Peptide and nickel transport system	M00349	Microcin C transport system	100	0
Phosphate and amino acid transport system	M00222	Phosphate transport system	100	0
	M00237	Branched-chain amino acid transport system	100	0
Purine metabolism	M00048	Inosine monophosphate biosynthesis, PRPP + glutamine => IMP	100	0
	M00049	Adenine ribonucleotide biosynthesis, IMP => ADP,ATP	100	0
	M00050	Guanine ribonucleotide biosynthesis IMP => GDP,GTP	100	0
Pyrimidine metabolism	M00052	Pyrimidine ribonucleotide biosynthesis, UMP => UDP/UTP,CDP/CTP	100	0

RNA polymerase	M00183	RNA polymerase, bacteria	100	0
Saccharide, polyol, and lipid transport system	M00210	Phospholipid transport system	100	0
Serine and threonine metabolism	M00018	Threonine biosynthesis, aspartate => homoserine => threonine	100	0
Sulfur metabolism	M00596	Dissimilatory sulfate reduction, sulfate => H2S	100	0
Two-component regulatory system	M00434	PhoR-PhoB (phosphate starvation response) two-component regulatory system	100	0
	M00450	BaeS-BaeR (envelope stress response) two-component regulatory system	100	0
	M00453	QseC-QseB (quorum sensing) two-component regulatory system	100	0
	M00475	BarA-UvrY (central carbon metabolism) two-component regulatory system	100	0
	M00493	AlgZ-AlgR (alginate production) two-component regulatory system	100	0
	M00501	PilS-PilR (type 4 fimbriae synthesis) two-component regulatory system	100	0
Genes under selection in treatment sediment identified by Tajima's test of neutrality				
Fatty acid metabolism	M00086	beta-Oxidation, acyl-CoA synthesis	100	0
Phosphotransferase system (PTS)	M00273	PTS system, fructose-specific II component	100	0

[a] MCR is the module completion ratio calculated for each functional module defined by the Kyoto Encyclopedia of Genes and Genomes.

[b] Q value is calculated based on the P values, indicating the probability that a reaction module was identified by chance. Q values less than 0.05 were considered significant.

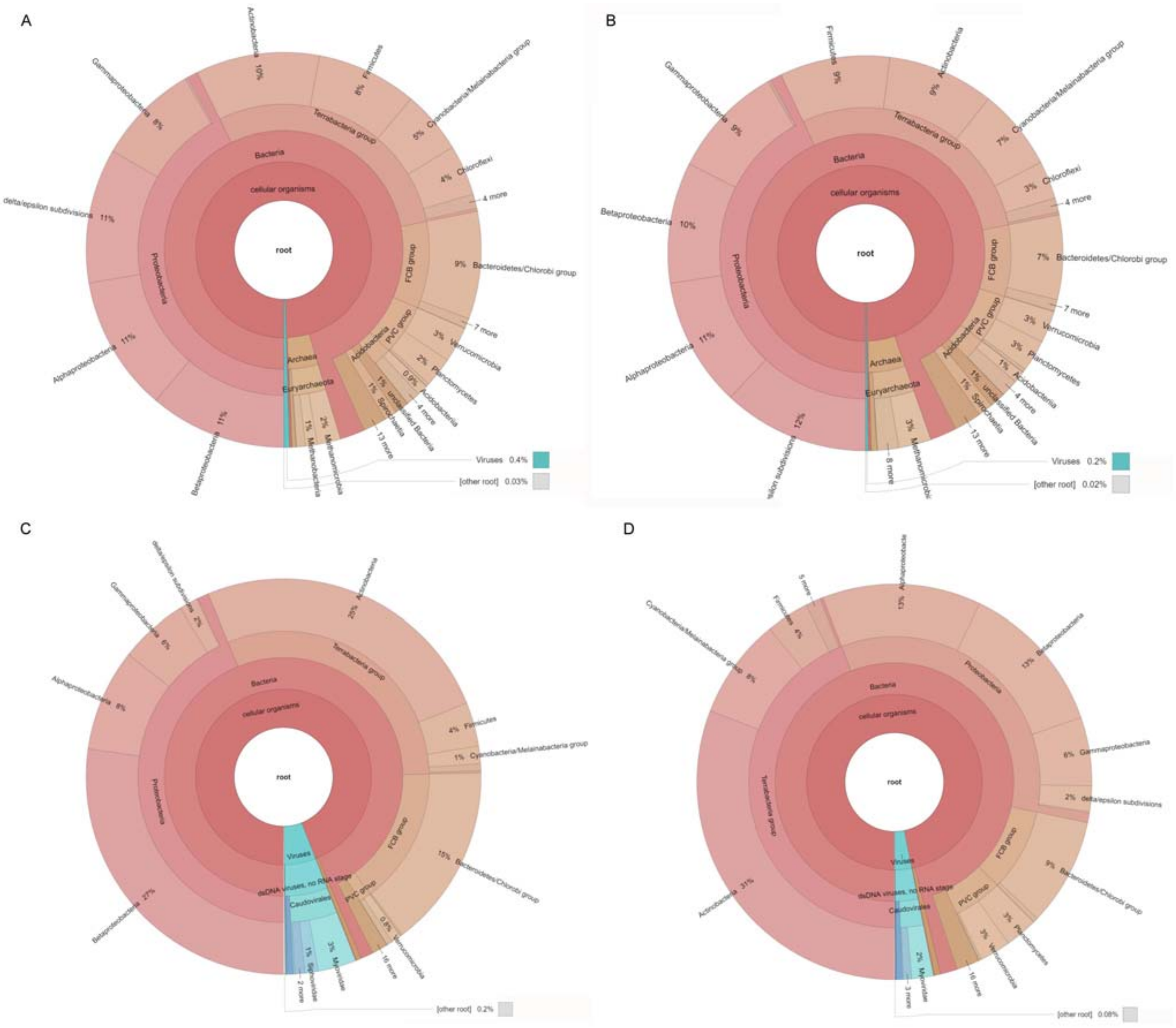

Supplemental Figure S1. Taxonomic distribution of (A) treatment-sediment sample, (B) control-sediment sample, (C) treatment-water sample, (D) control-water sample.

Supplementary Dataset S1. Significantly differentially abundant genera between water samples collected from the treatment IPRS system and control water reservoir. logFC, log2-transformed fold change; logCPM, log2-transformed counts per million. FDR P value < 0.05 was significant.

Superkingdom	Genus	logFC	logCPM	P-value	FDR P-value
Archaea	Picrophilus	1.43E+00	5.65E+00	1.19E-04	9.54E-04
Archaea	Staphylothermus	1.36E+00	5.15E+00	3.04E-04	2.21E-03
Archaea	Desulfurococcus	1.21E+00	5.58E+00	1.06E-03	6.32E-03
Archaea	Methanothermus	1.20E+00	5.10E+00	1.43E-03	8.07E-03
Archaea	Caldivirga	1.17E+00	5.51E+00	1.66E-03	8.93E-03
Archaea	Candidatus_Nitrosoarchaeum	9.18E-01	6.00E+00	1.20E-02	4.52E-02
Archaea	Candidatus_Methanomethylophilus	-9.51E-01	4.30E+00	1.36E-02	4.97E-02
Archaea	Candidatus_Korarchaeum	-9.68E-01	4.89E+00	1.02E-02	3.94E-02
Archaea	Natronococcus	-9.69E-01	5.72E+00	8.53E-03	3.51E-02
Archaea	Methanofollis	-9.80E-01	5.49E+00	8.09E-03	3.41E-02
Archaea	Ignicoccus	-1.02E+00	3.92E+00	9.64E-03	3.80E-02
Archaea	Methanopyrus	-1.03E+00	4.59E+00	6.86E-03	2.98E-02
Archaea	Fervidicoccus	-1.06E+00	3.66E+00	8.38E-03	3.46E-02
Archaea	Haloferax	-1.08E+00	5.97E+00	3.17E-03	1.52E-02
Archaea	Halapricum	-1.10E+00	5.01E+00	3.38E-03	1.59E-02
Archaea	Candidatus_Caldiarchaeum	-1.14E+00	5.45E+00	2.21E-03	1.13E-02
Archaea	Halalkalicoccus	-1.14E+00	4.14E+00	3.36E-03	1.58E-02
Archaea	Aeropyrum	-1.15E+00	4.79E+00	2.41E-03	1.21E-02
Archaea	Geoglobus	-1.19E+00	4.50E+00	1.88E-03	9.88E-03
Archaea	Methermicoccus	-1.20E+00	4.71E+00	1.58E-03	8.71E-03
Archaea	Haladaptatus	-1.27E+00	5.67E+00	6.51E-04	4.25E-03
Archaea	Halomicrobium	-1.27E+00	4.53E+00	9.17E-04	5.62E-03
Archaea	Halobiforma	-1.27E+00	4.36E+00	9.93E-04	5.99E-03
Archaea	Hyperthermus	-1.31E+00	3.81E+00	1.04E-03	6.21E-03
Archaea	Halorhabdus	-1.31E+00	5.04E+00	4.91E-04	3.30E-03
Archaea	Halolamina	-1.32E+00	4.37E+00	6.77E-04	4.37E-03
Archaea	Haloplanus	-1.32E+00	4.38E+00	6.62E-04	4.30E-03
Archaea	Thermofilum	-1.39E+00	5.26E+00	2.13E-04	1.62E-03
Archaea	Candidatus_Halobonum	-1.68E+00	4.68E+00	1.34E-05	1.41E-04
Archaea	Thermosphaera	-1.76E+00	3.40E+00	2.55E-05	2.43E-04
Bacteria	Emticicia	3.92E+00	1.28E+01	9.05E-23	2.06E-20
Bacteria	Rhodoluna	3.64E+00	1.39E+01	2.40E-20	3.18E-18
Bacteria	Neorhizobium	3.46E+00	8.33E+00	1.50E-18	1.50E-16
Bacteria	Flectobacillus	3.22E+00	1.19E+01	7.98E-17	6.06E-15
Bacteria	Polynucleobacter	2.88E+00	1.51E+01	3.65E-14	1.87E-12
Bacteria	Candidatus_Aquiluna	2.65E+00	1.19E+01	2.18E-12	8.49E-11
Bacteria	Cetobacterium	2.63E+00	8.11E+00	3.64E-12	1.38E-10
Bacteria	Escherichia	2.40E+00	8.25E+00	1.51E-10	3.83E-09
Bacteria	Cellulosilyticum	2.22E+00	7.84E+00	2.56E-09	5.45E-08
Bacteria	Limnohabitans	2.21E+00	1.42E+01	2.54E-09	5.45E-08
Bacteria	Avibacterium	2.16E+00	7.26E+00	8.25E-09	1.60E-07
Bacteria	Terrisporobacter	2.16E+00	7.09E+00	8.74E-09	1.66E-07
Bacteria	Novosphingobium	2.12E+00	1.28E+01	1.01E-08	1.89E-07

Bacteria	Candidatus_Hodgkinia	1.97E+00	4.76E+00	4.04E-07	6.02E-06
Bacteria	Sediminibacterium	1.97E+00	1.33E+01	8.69E-08	1.41E-06
Bacteria	Riemerella	1.94E+00	9.30E+00	1.35E-07	2.13E-06
Bacteria	Phaeobacter	1.90E+00	7.32E+00	2.85E-07	4.37E-06
Bacteria	Salmonella	1.88E+00	7.16E+00	3.78E-07	5.68E-06
Bacteria	Nitratiruptor	1.87E+00	7.18E+00	4.43E-07	6.47E-06
Bacteria	Candidatus_Tremblaya	1.80E+00	4.91E+00	3.07E-06	3.79E-05
Bacteria	Methylovorus	1.79E+00	1.08E+01	9.53E-07	1.31E-05
Bacteria	Rathayibacter	1.79E+00	8.87E+00	1.03E-06	1.38E-05
Bacteria	Erythrobacter	1.79E+00	1.09E+01	9.79E-07	1.32E-05
Bacteria	Asinibacterium	1.71E+00	1.28E+01	2.91E-06	3.65E-05
Bacteria	Cronobacter	1.67E+00	7.40E+00	5.28E-06	6.23E-05
Bacteria	Glaciibacter	1.66E+00	9.37E+00	5.10E-06	6.07E-05
Bacteria	Methylobacillus	1.65E+00	1.09E+01	5.69E-06	6.67E-05
Bacteria	Paraclostridium	1.65E+00	6.15E+00	9.24E-06	1.00E-04
Bacteria	Mycetocola	1.64E+00	9.24E+00	6.70E-06	7.79E-05
Bacteria	Cryobacterium	1.64E+00	9.23E+00	7.30E-06	8.39E-05
Bacteria	Ralstonia	1.63E+00	1.28E+01	7.38E-06	8.40E-05
Bacteria	Fangia	1.63E+00	8.36E+00	8.35E-06	9.37E-05
Bacteria	Leadbetterella	1.63E+00	9.99E+00	8.02E-06	9.06E-05
Bacteria	Photorhabdus	1.62E+00	8.71E+00	8.44E-06	9.41E-05
Bacteria	Citromicrobium	1.62E+00	8.75E+00	9.12E-06	9.96E-05
Bacteria	Leifsonia	1.62E+00	1.08E+01	8.91E-06	9.79E-05
Bacteria	Trabulsiella	1.58E+00	6.09E+00	1.97E-05	1.95E-04
Bacteria	Sphingopyxis	1.58E+00	1.05E+01	1.39E-05	1.45E-04
Bacteria	Romboutsia	1.57E+00	5.95E+00	2.56E-05	2.43E-04
Bacteria	Agromyces	1.56E+00	9.93E+00	1.68E-05	1.68E-04
Bacteria	Leucobacter	1.56E+00	1.08E+01	1.68E-05	1.68E-04
Bacteria	Eremococcus	1.56E+00	6.12E+00	2.56E-05	2.43E-04
Bacteria	Porphyrobacter	1.55E+00	9.96E+00	1.97E-05	1.95E-04
Bacteria	Clavibacter	1.54E+00	9.44E+00	2.25E-05	2.20E-04
Bacteria	Succinatimonas	1.54E+00	7.37E+00	2.64E-05	2.49E-04
Bacteria	Runella	1.49E+00	1.16E+01	4.17E-05	3.71E-04
Bacteria	Pandoraea	1.48E+00	1.12E+01	4.49E-05	3.95E-04
Bacteria	Gaetbulibacter	1.47E+00	6.55E+00	6.59E-05	5.68E-04
Bacteria	Chryseobacterium	1.44E+00	1.18E+01	6.65E-05	5.70E-04
Bacteria	Laribacter	1.42E+00	9.24E+00	8.55E-05	7.18E-04
Bacteria	Blastomonas	1.41E+00	9.05E+00	1.01E-04	8.36E-04
Bacteria	Salinibacterium	1.40E+00	8.20E+00	1.17E-04	9.48E-04
Bacteria	Kordia	1.40E+00	8.42E+00	1.18E-04	9.48E-04
Bacteria	Aminobacter	1.39E+00	8.58E+00	1.20E-04	9.58E-04
Bacteria	Methylotenera	1.37E+00	1.21E+01	1.55E-04	1.20E-03
Bacteria	Elizabethkingia	1.36E+00	9.14E+00	1.67E-04	1.28E-03
Bacteria	Dechloromonas	1.33E+00	1.08E+01	2.25E-04	1.70E-03
Bacteria	Epilithonimonas	1.33E+00	8.90E+00	2.48E-04	1.86E-03
Bacteria	Turicibacter	1.30E+00	6.68E+00	3.91E-04	2.72E-03
Bacteria	Lachnobacterium	1.30E+00	5.10E+00	5.71E-04	3.79E-03

Bacteria	Kozakia	1.29E+00	7.43E+00	3.69E-04	2.59E-03
Bacteria	Sutterella	1.28E+00	9.87E+00	3.72E-04	2.60E-03
Bacteria	Candidatus_Puniceispirillum	1.28E+00	8.98E+00	4.03E-04	2.80E-03
Bacteria	Collimonas	1.27E+00	1.09E+01	4.26E-04	2.93E-03
Bacteria	Lactococcus	1.27E+00	8.11E+00	4.54E-04	3.08E-03
Bacteria	Gryllotalpicola	1.27E+00	9.06E+00	4.40E-04	3.00E-03
Bacteria	Cupriavidus	1.27E+00	1.23E+01	4.28E-04	2.93E-03
Bacteria	Candidatus_Carsonella	1.26E+00	5.27E+00	7.35E-04	4.69E-03
Bacteria	Dyadobacter	1.26E+00	1.13E+01	4.78E-04	3.23E-03
Bacteria	Fibrella	1.25E+00	9.94E+00	5.50E-04	3.67E-03
Bacteria	Fibrisoma	1.24E+00	9.84E+00	6.05E-04	3.99E-03
Bacteria	Humibacter	1.23E+00	9.00E+00	6.39E-04	4.19E-03
Bacteria	Paeniclostridium	1.23E+00	5.51E+00	9.28E-04	5.67E-03
Bacteria	Candidatus_Regiella	1.22E+00	6.63E+00	8.59E-04	5.29E-03
Bacteria	Rhodobacter	1.21E+00	1.04E+01	7.73E-04	4.87E-03
Bacteria	Delftia	1.20E+00	1.06E+01	8.34E-04	5.17E-03
Bacteria	Methylophilus	1.20E+00	1.06E+01	8.41E-04	5.20E-03
Bacteria	Alysiella	1.20E+00	7.13E+00	9.68E-04	5.89E-03
Bacteria	Candidatus_Zinderia	1.19E+00	4.39E+00	2.01E-03	1.04E-02
Bacteria	Deefgea	1.18E+00	7.75E+00	1.11E-03	6.55E-03
Bacteria	Candidatus_Profftella	1.17E+00	6.07E+00	1.38E-03	7.83E-03
Bacteria	Haemophilus	1.17E+00	8.56E+00	1.23E-03	7.17E-03
Bacteria	Clostridioides	1.16E+00	7.50E+00	1.34E-03	7.72E-03
Bacteria	Azospira	1.16E+00	9.89E+00	1.28E-03	7.43E-03
Bacteria	Klebsiella	1.15E+00	6.89E+00	1.53E-03	8.55E-03
Bacteria	Helcococcus	1.15E+00	6.85E+00	1.55E-03	8.61E-03
Bacteria	Anaerostipes	1.15E+00	6.91E+00	1.61E-03	8.75E-03
Bacteria	Phaseolibacter	1.14E+00	6.04E+00	1.82E-03	9.64E-03
Bacteria	Pelistega	1.14E+00	8.59E+00	1.56E-03	8.61E-03
Bacteria	Spirosoma	1.14E+00	1.15E+01	1.52E-03	8.55E-03
Bacteria	Myroides	1.14E+00	9.57E+00	1.55E-03	8.61E-03
Bacteria	Parasutterella	1.14E+00	9.18E+00	1.59E-03	8.74E-03
Bacteria	Flavobacterium	1.13E+00	1.40E+01	1.61E-03	8.75E-03
Bacteria	Arcobacter	1.13E+00	8.84E+00	1.75E-03	9.39E-03
Bacteria	Taylorella	1.13E+00	8.93E+00	1.75E-03	9.39E-03
Bacteria	Herminiimonas	1.12E+00	9.82E+00	1.81E-03	9.64E-03
Bacteria	Agrococcus	1.12E+00	8.96E+00	1.88E-03	9.88E-03
Bacteria	Yersinia	1.11E+00	8.74E+00	2.15E-03	1.11E-02
Bacteria	Lachnoanaerobaculum	1.10E+00	5.33E+00	3.13E-03	1.50E-02
Bacteria	Basilea	1.10E+00	8.25E+00	2.36E-03	1.19E-02
Bacteria	Oxalobacter	1.10E+00	1.03E+01	2.29E-03	1.16E-02
Bacteria	Marvinbryantia	1.09E+00	6.47E+00	2.77E-03	1.36E-02
Bacteria	Simonsiella	1.09E+00	6.59E+00	2.83E-03	1.38E-02
Bacteria	Aliivibrio	1.09E+00	6.67E+00	2.85E-03	1.38E-02
Bacteria	Azovibrio	1.08E+00	9.59E+00	2.65E-03	1.31E-02
Bacteria	Solibacillus	1.07E+00	5.88E+00	3.43E-03	1.60E-02
Bacteria	Bacteroides	1.07E+00	1.18E+01	2.91E-03	1.40E-02

Bacteria	Rudanella	1.06E+00	9.86E+00	3.24E-03	1.54E-02
Bacteria	Brachymonas	1.05E+00	1.02E+01	3.31E-03	1.57E-02
Bacteria	Gulosibacter	1.05E+00	8.62E+00	3.39E-03	1.59E-02
Bacteria	Neisseria	1.05E+00	1.02E+01	3.50E-03	1.63E-02
Bacteria	Acidovorax	1.04E+00	1.31E+01	3.82E-03	1.76E-02
Bacteria	Comamonas	1.03E+00	1.24E+01	3.93E-03	1.81E-02
Bacteria	Hydrogenovibrio	1.03E+00	6.19E+00	5.09E-03	2.27E-02
Bacteria	Bizionia	1.02E+00	7.09E+00	4.90E-03	2.22E-02
Bacteria	Peptoclostridium	1.01E+00	7.68E+00	4.91E-03	2.22E-02
Bacteria	Alicycliphilus	1.01E+00	1.00E+01	4.95E-03	2.22E-02
Bacteria	Curtobacterium	9.90E-01	9.28E+00	5.82E-03	2.57E-02
Bacteria	Catellicoccus	9.89E-01	4.94E+00	8.23E-03	3.43E-02
Bacteria	Citrobacter	9.85E-01	7.66E+00	6.28E-03	2.76E-02
Bacteria	Sodalis	9.75E-01	6.58E+00	7.35E-03	3.17E-02
Bacteria	Curvibacter	9.73E-01	1.16E+01	6.57E-03	2.88E-02
Bacteria	Enhydrobacter	9.72E-01	6.80E+00	7.35E-03	3.17E-02
Bacteria	Halobacteriovorax	9.61E-01	8.30E+00	7.51E-03	3.22E-02
Bacteria	Pseudoclavibacter	9.60E-01	9.54E+00	7.41E-03	3.18E-02
Bacteria	Candidatus_Hamiltonella	9.56E-01	6.39E+00	8.73E-03	3.54E-02
Bacteria	Simplicispira	9.48E-01	9.98E+00	8.18E-03	3.42E-02
Bacteria	Methylobacter	9.39E-01	8.65E+00	8.89E-03	3.58E-02
Bacteria	Moraxella	9.36E-01	8.79E+00	9.13E-03	3.67E-02
Bacteria	Weeksella	9.32E-01	8.75E+00	9.39E-03	3.74E-02
Bacteria	Microbacterium	9.27E-01	1.23E+01	9.56E-03	3.78E-02
Bacteria	Bibersteinia	9.26E-01	5.44E+00	1.22E-02	4.57E-02
Bacteria	Leptotrichia	9.20E-01	8.07E+00	1.05E-02	4.07E-02
Bacteria	Vitreoscilla	9.16E-01	7.55E+00	1.10E-02	4.25E-02
Bacteria	Siccibacter	9.11E-01	5.83E+00	1.29E-02	4.77E-02
Bacteria	Gallionella	9.04E-01	9.18E+00	1.17E-02	4.43E-02
Bacteria	Bacteriovorax	8.99E-01	1.01E+01	1.20E-02	4.52E-02
Bacteria	Candidatus_Schmidhempelia	8.98E-01	6.38E+00	1.37E-02	4.98E-02
Bacteria	Hydrogenophaga	8.97E-01	1.11E+01	1.21E-02	4.54E-02
Bacteria	Candidatus_Glomeribacter	8.96E-01	8.44E+00	1.26E-02	4.66E-02
Bacteria	Clostridium	8.93E-01	1.20E+01	1.25E-02	4.66E-02
Bacteria	Burkholderia	8.92E-01	1.25E+01	1.26E-02	4.66E-02
Bacteria	Janthinobacterium	8.86E-01	1.16E+01	1.32E-02	4.85E-02
Bacteria	Desulfocapsa	-8.97E-01	6.91E+00	1.31E-02	4.83E-02
Bacteria	Desulfitibacter	-8.99E-01	6.58E+00	1.32E-02	4.85E-02
Bacteria	Arenimonas	-8.99E-01	9.50E+00	1.20E-02	4.52E-02
Bacteria	Syntrophothermus	-9.03E-01	6.53E+00	1.29E-02	4.77E-02
Bacteria	Thiorhodovibrio	-9.04E-01	8.36E+00	1.18E-02	4.48E-02
Bacteria	Maricaulis	-9.05E-01	7.43E+00	1.20E-02	4.52E-02
Bacteria	Parvularcula	-9.07E-01	8.07E+00	1.17E-02	4.43E-02
Bacteria	Chamaesiphon	-9.10E-01	7.95E+00	1.14E-02	4.38E-02
Bacteria	Heliobacterium	-9.11E-01	7.12E+00	1.17E-02	4.43E-02
Bacteria	Desulfonatronospira	-9.16E-01	6.80E+00	1.15E-02	4.39E-02
Bacteria	Simkania	-9.25E-01	7.86E+00	1.01E-02	3.94E-02

Bacteria	Geothrix	-9.28E-01	8.10E+00	9.86E-03	3.86E-02
Bacteria	Waddlia	-9.31E-01	7.06E+00	9.99E-03	3.90E-02
Bacteria	Fortiea	-9.33E-01	6.81E+00	1.00E-02	3.90E-02
Bacteria	Akkermansia	-9.33E-01	8.75E+00	9.32E-03	3.72E-02
Bacteria	Orientia	-9.34E-01	7.19E+00	9.73E-03	3.83E-02
Bacteria	Congregibacter	-9.39E-01	7.27E+00	9.31E-03	3.72E-02
Bacteria	Myxococcus	-9.42E-01	9.93E+00	8.56E-03	3.52E-02
Bacteria	Candidatus_Phaeomarinobacter	-9.44E-01	8.09E+00	8.70E-03	3.54E-02
Bacteria	Skermanella	-9.47E-01	9.08E+00	8.30E-03	3.45E-02
Bacteria	Rhodospirillum	-9.48E-01	9.16E+00	8.26E-03	3.44E-02
Bacteria	Desulfurispirillum	-9.50E-01	6.97E+00	8.63E-03	3.53E-02
Bacteria	Thermanaerovibrio	-9.51E-01	6.75E+00	8.71E-03	3.54E-02
Bacteria	Candidatus_Midichloria	-9.51E-01	6.50E+00	8.88E-03	3.58E-02
Bacteria	Desulfurispora	-9.53E-01	6.43E+00	8.81E-03	3.56E-02
Bacteria	Desulfomonile	-9.53E-01	7.88E+00	8.11E-03	3.41E-02
Bacteria	Roseiflexus	-9.55E-01	1.04E+01	7.65E-03	3.26E-02
Bacteria	Prosthecochloris	-9.56E-01	6.70E+00	8.38E-03	3.46E-02
Bacteria	Granulicella	-9.59E-01	8.48E+00	7.59E-03	3.24E-02
Bacteria	Thermotoga	-9.65E-01	6.62E+00	7.90E-03	3.35E-02
Bacteria	Candidatus_Endolissoclinum	-9.74E-01	5.97E+00	7.91E-03	3.35E-02
Bacteria	Halonatronum	-9.76E-01	5.84E+00	7.94E-03	3.36E-02
Bacteria	Woodsholea	-9.82E-01	6.94E+00	6.70E-03	2.93E-02
Bacteria	Rhodovibrio	-9.87E-01	8.00E+00	6.14E-03	2.70E-02
Bacteria	Dethiosulfovibrio	-9.88E-01	5.98E+00	7.10E-03	3.08E-02
Bacteria	Levyella	-9.88E-01	4.92E+00	8.64E-03	3.53E-02
Bacteria	Candidatus_Atelocyanobacterium	-1.01E+00	6.73E+00	5.46E-03	2.42E-02
Bacteria	Desulfohalobium	-1.01E+00	6.39E+00	5.56E-03	2.46E-02
Bacteria	Tistrella	-1.01E+00	8.01E+00	4.97E-03	2.22E-02
Bacteria	Halothece	-1.02E+00	7.10E+00	4.93E-03	2.22E-02
Bacteria	Acaryochloris	-1.02E+00	7.83E+00	4.74E-03	2.15E-02
Bacteria	Desulfobacca	-1.02E+00	7.26E+00	4.73E-03	2.15E-02
Bacteria	Thermosinus	-1.03E+00	6.57E+00	4.69E-03	2.14E-02
Bacteria	Cyanothece	-1.04E+00	1.05E+01	3.72E-03	1.72E-02
Bacteria	Syntrophobacter	-1.05E+00	7.61E+00	3.68E-03	1.71E-02
Bacteria	Candidatus_Moranella	-1.05E+00	3.38E+00	1.02E-02	3.94E-02
Bacteria	Kallipyga	-1.06E+00	4.75E+00	5.21E-03	2.32E-02
Bacteria	Thermincola	-1.06E+00	6.41E+00	3.68E-03	1.71E-02
Bacteria	Xenococcus	-1.06E+00	7.75E+00	3.25E-03	1.55E-02
Bacteria	Candidatus_Saccharimonas	-1.06E+00	7.22E+00	3.29E-03	1.56E-02
Bacteria	Leptolyngbya	-1.07E+00	1.07E+01	2.88E-03	1.39E-02
Bacteria	Rubrobacter	-1.08E+00	8.83E+00	2.77E-03	1.36E-02
Bacteria	Pelobacter	-1.08E+00	8.97E+00	2.74E-03	1.35E-02
Bacteria	Hyphomonas	-1.08E+00	1.04E+01	2.60E-03	1.29E-02
Bacteria	Jannaschia	-1.08E+00	6.83E+00	2.85E-03	1.38E-02
Bacteria	Dehalogenimonas	-1.08E+00	6.79E+00	2.84E-03	1.38E-02
Bacteria	Enhygromyxa	-1.09E+00	8.69E+00	2.44E-03	1.22E-02
Bacteria	Oceanithermus	-1.10E+00	7.11E+00	2.51E-03	1.26E-02

Bacteria	Parvibaculum	-1.10E+00	8.27E+00	2.27E-03	1.15E-02
Bacteria	Candidatus_Desulforudis	-1.12E+00	6.65E+00	2.17E-03	1.11E-02
Bacteria	Thermomicrobium	-1.13E+00	7.30E+00	1.90E-03	9.95E-03
Bacteria	Ammonifex	-1.13E+00	6.41E+00	2.05E-03	1.06E-02
Bacteria	Halothermothrix	-1.15E+00	6.61E+00	1.66E-03	8.93E-03
Bacteria	Geminicoccus	-1.15E+00	8.45E+00	1.40E-03	7.96E-03
Bacteria	Caldilinea	-1.16E+00	9.07E+00	1.34E-03	7.72E-03
Bacteria	Rubidibacter	-1.16E+00	7.33E+00	1.37E-03	7.80E-03
Bacteria	Diplosphaera	-1.16E+00	9.37E+00	1.23E-03	7.17E-03
Bacteria	Thermus	-1.16E+00	9.28E+00	1.23E-03	7.17E-03
Bacteria	Coriobacterium	-1.18E+00	5.18E+00	1.65E-03	8.93E-03
Bacteria	Mastigocladopsis	-1.18E+00	7.71E+00	1.10E-03	6.52E-03
Bacteria	Kyrpidia	-1.20E+00	7.00E+00	9.71E-04	5.89E-03
Bacteria	Anaeroglobus	-1.20E+00	4.98E+00	1.42E-03	8.00E-03
Bacteria	Tepidicaulis	-1.21E+00	7.96E+00	8.17E-04	5.11E-03
Bacteria	Stigmatella	-1.21E+00	8.35E+00	7.96E-04	5.00E-03
Bacteria	Candidatus_Omnitrophus	-1.21E+00	7.46E+00	8.22E-04	5.12E-03
Bacteria	Prochlorococcus	-1.22E+00	1.14E+01	7.24E-04	4.65E-03
Bacteria	Thermogemmatispora	-1.22E+00	7.97E+00	7.42E-04	4.71E-03
Bacteria	Desulfonatronum	-1.23E+00	7.47E+00	7.28E-04	4.66E-03
Bacteria	Gloeocapsa	-1.23E+00	9.55E+00	6.71E-04	4.35E-03
Bacteria	Acidocella	-1.24E+00	7.65E+00	5.97E-04	3.95E-03
Bacteria	Nitrococcus	-1.26E+00	7.65E+00	5.10E-04	3.42E-03
Bacteria	Candidatus_Pelagibacter	-1.29E+00	1.09E+01	3.55E-04	2.50E-03
Bacteria	Thalassobaculum	-1.29E+00	8.26E+00	3.53E-04	2.50E-03
Bacteria	Syntrophomonas	-1.30E+00	6.33E+00	4.05E-04	2.80E-03
Bacteria	Corallococcus	-1.31E+00	8.26E+00	3.13E-04	2.23E-03
Bacteria	Rhodothermus	-1.31E+00	8.45E+00	3.09E-04	2.23E-03
Bacteria	Opitutus	-1.31E+00	1.06E+01	2.88E-04	2.14E-03
Bacteria	Lentisphaera	-1.31E+00	8.75E+00	2.92E-04	2.14E-03
Bacteria	Candidatus_Koribacter	-1.31E+00	8.36E+00	2.91E-04	2.14E-03
Bacteria	Desulfarculus	-1.31E+00	7.27E+00	3.08E-04	2.23E-03
Bacteria	Acidiphilium	-1.32E+00	8.89E+00	2.71E-04	2.01E-03
Bacteria	Pseudohaliea	-1.33E+00	7.65E+00	2.53E-04	1.90E-03
Bacteria	Desulfatiglans	-1.35E+00	7.34E+00	2.02E-04	1.54E-03
Bacteria	Chloroflexus	-1.36E+00	1.00E+01	1.68E-04	1.28E-03
Bacteria	Candidatus_Methylomirabilis	-1.37E+00	8.06E+00	1.60E-04	1.24E-03
Bacteria	Gemmatirosa	-1.37E+00	9.00E+00	1.52E-04	1.19E-03
Bacteria	Methylocystis	-1.38E+00	1.06E+01	1.38E-04	1.09E-03
Bacteria	Dethiobacter	-1.39E+00	6.60E+00	1.52E-04	1.19E-03
Bacteria	Granulibacter	-1.40E+00	7.48E+00	1.22E-04	9.66E-04
Bacteria	Silanimonas	-1.40E+00	8.04E+00	1.17E-04	9.48E-04
Bacteria	Hirschia	-1.40E+00	8.42E+00	1.12E-04	9.19E-04
Bacteria	Caulobacter	-1.40E+00	1.07E+01	1.03E-04	8.47E-04
Bacteria	Thermobaculum	-1.43E+00	8.16E+00	8.64E-05	7.21E-04
Bacteria	Meiothermus	-1.44E+00	1.01E+01	7.31E-05	6.20E-04
Bacteria	Pedosphaera	-1.44E+00	1.16E+01	6.60E-05	5.68E-04

Bacteria	Reyranella	-1.47E+00	9.78E+00	5.12E-05	4.48E-04
Bacteria	Ponticaulis	-1.47E+00	7.59E+00	5.32E-05	4.63E-04
Bacteria	Thermacetogenium	-1.50E+00	6.61E+00	4.41E-05	3.90E-04
Bacteria	Desulfurivibrio	-1.50E+00	7.06E+00	4.07E-05	3.64E-04
Bacteria	Symbiobacterium	-1.51E+00	7.30E+00	3.63E-05	3.27E-04
Bacteria	Gloeobacter	-1.51E+00	8.90E+00	3.16E-05	2.90E-04
Bacteria	Sphaerobacter	-1.51E+00	8.13E+00	3.21E-05	2.91E-04
Bacteria	Chthonomonas	-1.52E+00	8.38E+00	3.04E-05	2.80E-04
Bacteria	Truepera	-1.52E+00	7.60E+00	3.20E-05	2.91E-04
Bacteria	Cystobacter	-1.52E+00	8.85E+00	2.87E-05	2.68E-04
Bacteria	Thermorudis	-1.55E+00	7.57E+00	2.20E-05	2.16E-04
Bacteria	Anaeromyxobacter	-1.57E+00	9.78E+00	1.51E-05	1.54E-04
Bacteria	Plesiocystis	-1.58E+00	8.85E+00	1.46E-05	1.51E-04
Bacteria	Thermosynechococcus	-1.58E+00	7.78E+00	1.47E-05	1.51E-04
Bacteria	Thermaerobacter	-1.59E+00	8.12E+00	1.35E-05	1.41E-04
Bacteria	Coraliomargarita	-1.63E+00	9.58E+00	7.32E-06	8.39E-05
Bacteria	Candidatus_Solibacter	-1.67E+00	9.80E+00	4.83E-06	5.78E-05
Bacteria	Nodosilinea	-1.70E+00	8.03E+00	3.28E-06	3.99E-05
Bacteria	Haliangium	-1.72E+00	9.02E+00	2.64E-06	3.34E-05
Bacteria	Oscillochloris	-1.75E+00	9.01E+00	1.73E-06	2.24E-05
Bacteria	Thermoanaerobaculum	-1.76E+00	7.71E+00	1.67E-06	2.19E-05
Bacteria	Prochlorothrix	-1.76E+00	8.06E+00	1.56E-06	2.05E-05
Bacteria	Zavarzinella	-1.81E+00	1.11E+01	7.70E-07	1.10E-05
Bacteria	Sorangium	-1.98E+00	1.01E+01	7.80E-08	1.28E-06
Bacteria	Roseomonas	-2.11E+00	1.04E+01	1.16E-08	2.13E-07
Bacteria	Patulibacter	-2.12E+00	9.57E+00	1.07E-08	1.98E-07
Bacteria	Acidimicrobium	-2.13E+00	9.28E+00	8.51E-09	1.63E-07
Bacteria	Synechocystis	-2.14E+00	9.59E+00	8.07E-09	1.59E-07
Bacteria	Ferrimicrobium	-2.15E+00	9.37E+00	7.00E-09	1.39E-07
Bacteria	Chloracidobacterium	-2.19E+00	9.09E+00	3.94E-09	7.95E-08
Bacteria	Verrucomicrobium	-2.19E+00	1.23E+01	3.18E-09	6.50E-08
Bacteria	Conexibacter	-2.24E+00	1.04E+01	1.71E-09	3.73E-08
Bacteria	Chondromyces	-2.25E+00	9.47E+00	1.44E-09	3.18E-08
Bacteria	Elioraea	-2.28E+00	9.38E+00	9.57E-10	2.15E-08
Bacteria	Gemmata	-2.31E+00	1.07E+01	5.34E-10	1.22E-08
Bacteria	Bryobacter	-2.34E+00	9.37E+00	3.73E-10	8.75E-09
Bacteria	Phenylobacterium	-2.43E+00	9.43E+00	8.64E-11	2.37E-09
Bacteria	Gemmatimonas	-2.44E+00	1.08E+01	6.94E-11	1.94E-09
Bacteria	Rubritepida	-2.45E+00	1.01E+01	5.85E-11	1.70E-09
Bacteria	Isosphaera	-2.52E+00	9.88E+00	2.04E-11	6.37E-10
Bacteria	Phycisphaera	-2.52E+00	9.16E+00	2.08E-11	6.37E-10
Bacteria	Rhodopirellula	-2.53E+00	1.16E+01	1.66E-11	5.53E-10
Bacteria	Singulisphaera	-2.60E+00	1.07E+01	4.78E-12	1.77E-10
Bacteria	Candidatus_Microthrix	-2.70E+00	1.15E+01	9.15E-13	3.74E-11
Bacteria	Ilumatobacter	-2.81E+00	1.35E+01	1.23E-13	5.43E-12
Bacteria	Rubinisphaera	-2.82E+00	1.03E+01	1.21E-13	5.43E-12
Bacteria	Rickettsia	-2.84E+00	9.66E+00	8.14E-14	3.82E-12

Bacteria	Chthoniobacter	-2.85E+00	1.19E+01	7.03E-14	3.42E-12
Bacteria	Fimbriimonas	-2.85E+00	9.85E+00	7.08E-14	3.42E-12
Bacteria	Methylacidiphilum	-2.91E+00	9.77E+00	2.69E-14	1.43E-12
Bacteria	Gimesia	-2.91E+00	1.06E+01	2.24E-14	1.23E-12
Bacteria	Schlesneria	-3.07E+00	1.11E+01	1.36E-15	8.36E-14
Bacteria	Planctopirus	-3.12E+00	1.04E+01	4.98E-16	3.17E-14
Bacteria	Microcystis	-3.13E+00	1.28E+01	3.82E-16	2.54E-14
Bacteria	Blastopirellula	-3.14E+00	1.09E+01	3.65E-16	2.53E-14
Bacteria	Pirellula	-3.15E+00	1.14E+01	2.84E-16	2.06E-14
Bacteria	Belnapia	-3.42E+00	1.11E+01	1.76E-18	1.65E-16
Bacteria	Raphidiopsis	-3.45E+00	9.26E+00	1.23E-18	1.31E-16
Bacteria	Cylindrospermopsis	-3.64E+00	8.99E+00	3.18E-20	3.90E-18
Bacteria	Synechococcus	-4.51E+00	1.45E+01	4.10E-28	1.63E-25
Bacteria	Cyanobium	-6.78E+00	1.32E+01	6.63E-50	1.06E-46
Viruses	Orbivirus	1.12E+01	3.52E+00	1.16E-28	9.24E-26
Viruses	F116virus	5.12E+00	5.43E+00	2.07E-28	1.10E-25
Viruses	T7virus	4.18E+00	8.01E+00	1.45E-24	4.62E-22
Viruses	Bppunalikevirus	4.05E+00	8.71E+00	1.42E-23	3.77E-21
Viruses	Hepacivirus	5.28E+00	3.48E+00	2.43E-21	4.83E-19
Viruses	Cd119virus	6.00E+00	3.00E+00	1.31E-20	2.33E-18
Viruses	Betabaculovirus	-3.75E+00	7.06E+00	1.64E-20	2.62E-18
Viruses	Silviavirus	4.23E+00	4.49E+00	2.08E-20	3.02E-18
Viruses	Cp8virus	3.84E+00	5.39E+00	1.08E-19	1.23E-17
Viruses	Che8virus	3.65E+00	5.38E+00	2.59E-18	2.29E-16
Viruses	Sk1virus	4.41E+00	3.29E+00	2.58E-17	2.16E-15
Viruses	Phi29virus	4.61E+00	3.03E+00	5.34E-17	4.25E-15
Viruses	Cc31virus	3.30E+00	5.05E+00	1.64E-15	9.66E-14
Viruses	Che9cvirus	3.27E+00	4.47E+00	1.47E-14	8.36E-13
Viruses	Phikmvvirus	2.82E+00	6.62E+00	3.65E-13	1.57E-11
Viruses	P2virus	3.03E+00	4.40E+00	5.67E-13	2.38E-11
Viruses	Charlievirus	6.02E+00	1.59E+00	1.62E-12	6.47E-11
Viruses	Felixo1virus	2.75E+00	5.13E+00	5.76E-12	2.09E-10
Viruses	Epsilon15virus	3.47E+00	2.76E+00	8.60E-12	3.05E-10
Viruses	Ichnovirus	-2.97E+00	3.79E+00	1.10E-11	3.82E-10
Viruses	Xp10virus	2.62E+00	5.90E+00	1.60E-11	5.41E-10
Viruses	Phicbkvirus	2.58E+00	6.62E+00	1.78E-11	5.80E-10
Viruses	Bcep22likevirus	2.60E+00	5.88E+00	2.02E-11	6.37E-10
Viruses	Cp220virus	2.54E+00	6.60E+00	2.99E-11	9.00E-10
Viruses	Sp6virus	3.16E+00	3.05E+00	3.23E-11	9.54E-10
Viruses	Cafeteriavirus	-2.46E+00	7.65E+00	6.92E-11	1.94E-09
Viruses	P22virus	2.67E+00	4.31E+00	9.58E-11	2.59E-09
Viruses	N4virus	2.44E+00	7.52E+00	1.10E-10	2.91E-09
Viruses	Vi1virus	2.71E+00	4.05E+00	1.14E-10	2.97E-09
Viruses	Prymnesiovirus	-2.40E+00	9.02E+00	1.47E-10	3.77E-09
Viruses	Bignuzvirus	4.02E+00	1.83E+00	2.07E-10	5.15E-09
Viruses	Lambdavirus	2.47E+00	5.64E+00	2.10E-10	5.15E-09
Viruses	P1virus	2.60E+00	4.27E+00	2.84E-10	6.87E-09

Viruses	Bxz1virus	2.70E+00	3.70E+00	3.55E-10	8.43E-09
Viruses	Schizot4virus	2.33E+00	8.52E+00	4.73E-10	1.09E-08
Viruses	Sap6virus	2.42E+00	4.22E+00	3.14E-09	6.50E-08
Viruses	S16virus	2.53E+00	3.68E+00	3.18E-09	6.50E-08
Viruses	Cecivirus	3.54E+00	1.65E+00	1.29E-08	2.33E-07
Viruses	Hapunavirus	3.22E+00	1.92E+00	1.48E-08	2.65E-07
Viruses	Tm4virus	2.59E+00	2.97E+00	1.60E-08	2.84E-07
Viruses	T1virus	2.29E+00	4.24E+00	1.62E-08	2.84E-07
Viruses	Sp18virus	2.12E+00	5.39E+00	3.56E-08	6.17E-07
Viruses	Bcepmuvirus	2.63E+00	2.66E+00	3.87E-08	6.64E-07
Viruses	Bc431virus	2.11E+00	5.16E+00	4.83E-08	8.19E-07
Viruses	Biquartavirus	8.05E+00	5.19E-01	5.28E-08	8.86E-07
Viruses	Omegavirus	2.03E+00	6.05E+00	7.77E-08	1.28E-06
Viruses	Badnavirus	-2.20E+00	3.83E+00	1.08E-07	1.74E-06
Viruses	Twortvirus	2.05E+00	4.93E+00	1.26E-07	2.01E-06
Viruses	L5virus	1.93E+00	6.68E+00	2.31E-07	3.62E-06
Viruses	Macavirus	-3.27E+00	1.42E+00	2.64E-07	4.09E-06
Viruses	Tp21virus	4.24E+00	8.33E-01	3.22E-07	4.88E-06
Viruses	Potyvirus	3.37E+00	1.26E+00	4.16E-07	6.14E-06
Viruses	D3virus	1.94E+00	4.82E+00	5.42E-07	7.85E-06
Viruses	Rotavirus	3.19E+00	1.35E+00	5.84E-07	8.39E-06
Viruses	Caulimovirus	-3.76E+00	9.25E-01	8.12E-07	1.15E-05
Viruses	Se1virus	1.90E+00	4.90E+00	9.21E-07	1.29E-05
Viruses	Orthopoxvirus	-1.83E+00	6.35E+00	9.58E-07	1.31E-05
Viruses	Leporipoxvirus	-2.86E+00	1.61E+00	9.59E-07	1.31E-05
Viruses	Phikzvirus	1.83E+00	5.76E+00	1.16E-06	1.55E-05
Viruses	Jerseyvirus	2.25E+00	2.47E+00	2.31E-06	2.98E-05
Viruses	Yuavirus	1.77E+00	5.60E+00	2.64E-06	3.34E-05
Viruses	Muvirus	3.03E+00	1.22E+00	3.01E-06	3.74E-05
Viruses	Corndogvirus	-3.17E+00	1.09E+00	3.14E-06	3.85E-05
Viruses	Sfi21dt1virus	-2.90E+00	1.31E+00	3.68E-06	4.45E-05
Viruses	Phic31virus	1.83E+00	3.61E+00	8.70E-06	9.64E-05
Viruses	Phieco32virus	1.80E+00	3.83E+00	9.31E-06	1.00E-04
Viruses	Js98virus	1.72E+00	4.39E+00	1.14E-05	1.22E-04
Viruses	Cjw1virus	2.88E+00	1.09E+00	1.24E-05	1.32E-04
Viruses	K1gvirus	1.99E+00	2.58E+00	1.58E-05	1.60E-04
Viruses	Asfivirus	-1.94E+00	2.62E+00	2.30E-05	2.23E-04
Viruses	Chloriridovirus	1.58E+00	5.71E+00	2.42E-05	2.34E-04
Viruses	Iridovirus	1.53E+00	8.73E+00	2.74E-05	2.57E-04
Viruses	Cavemovirus	-7.26E+00	-1.66E-01	2.89E-05	2.68E-04
Viruses	Pbunavirus	1.50E+00	5.33E+00	6.91E-05	5.89E-04
Viruses	Phijl1virus	1.94E+00	2.10E+00	8.20E-05	6.92E-04
Viruses	Iltovirus	-2.37E+00	1.22E+00	9.89E-05	8.21E-04
Viruses	Bracovirus	-1.58E+00	2.83E+00	3.03E-04	2.21E-03
Viruses	Sp31virus	1.71E+00	2.26E+00	3.11E-04	2.23E-03
Viruses	Rhadinovirus	-1.75E+00	2.08E+00	3.54E-04	2.50E-03
Viruses	Cr3virus	1.30E+00	4.30E+00	7.48E-04	4.73E-03

Viruses	Scutavirus	-1.96E+00	1.17E+00	1.03E-03	6.19E-03
Viruses	Betaretrovirus	-3.17E+00	-4.59E-02	1.09E-03	6.47E-03
Viruses	Andromedavirus	1.27E+00	4.02E+00	1.25E-03	7.24E-03
Viruses	Marseillevirus	-1.32E+00	3.35E+00	1.36E-03	7.79E-03
Viruses	Influenzavirus_A	3.75E+00	-3.68E-01	1.60E-03	8.74E-03
Viruses	Phietavirus	1.24E+00	3.83E+00	1.81E-03	9.64E-03
Viruses	Megalocytivirus	1.23E+00	3.73E+00	1.98E-03	1.04E-02
Viruses	Gammaretrovirus	-1.85E+00	1.09E+00	2.13E-03	1.10E-02
Viruses	Lentivirus	-2.20E+00	5.58E-01	2.13E-03	1.10E-02
Viruses	Bronvirus	1.22E+00	3.72E+00	2.20E-03	1.12E-02
Viruses	Lymphocryptovirus	-3.01E+00	-1.66E-01	2.57E-03	1.28E-02
Viruses	Bcep78virus	1.10E+00	4.52E+00	3.96E-03	1.82E-02
Viruses	Varicellovirus	-1.12E+00	3.30E+00	6.82E-03	2.97E-02
Viruses	Suipoxvirus	1.11E+00	3.04E+00	8.15E-03	3.42E-02
Viruses	Alphacoronavirus	-2.74E+00	-3.68E-01	9.56E-03	3.78E-02
Viruses	Deltaretrovirus	-2.74E+00	-3.68E-01	9.56E-03	3.78E-02
Viruses	Ascovirus	9.50E-01	5.67E+00	9.86E-03	3.86E-02
Viruses	Cytomegalovirus	-1.15E+00	2.43E+00	1.15E-02	4.39E-02

Supplementary Dataset S2. Significantly differentially abundant genera between sediment samples collected from the treatment IPRS system and control water reservoir. logFC, log2-transformed fold change; logCPM, log2-transformed counts per million. FDR P value < 0.05 was significant.

**Superkingdom**	**Genus**	**logFC**	**logCPM**	**P-value**	**FDR P-value**
Archaea	Candidatus_Methanoperedens	-1.26E+00	1.04E+01	4.63E-04	4.79E-02
Bacteria	Dechloromonas	2.46E+00	1.23E+01	5.15E-11	2.67E-08
Bacteria	Microcystis	2.29E+00	1.37E+01	7.43E-10	2.31E-07
Bacteria	Pseudorhodobacter	1.75E+00	8.04E+00	1.73E-06	4.49E-04
Bacteria	Rhodobacter	1.68E+00	1.10E+01	3.88E-06	8.62E-04
Bacteria	Rhodoluna	1.49E+00	6.64E+00	4.56E-05	7.15E-03
Bacteria	Phycicoccus	1.25E+00	7.99E+00	5.59E-04	4.85E-02
Bacteria	Cyanothece	-1.48E+00	1.21E+01	4.60E-05	7.15E-03
Bacteria	Cyanobium	-2.70E+00	1.19E+01	8.88E-13	6.91E-10
Bacteria	Beggiatoa	-3.31E+00	1.16E+01	1.49E-17	2.32E-14
Viruses	Bppunalikevirus	2.41E+00	4.59E+00	5.59E-10	2.17E-07
Viruses	Xp10virus	1.84E+00	2.12E+00	2.74E-05	5.33E-03
Viruses	T7virus	1.62E+00	3.05E+00	5.48E-05	7.75E-03
Viruses	N4virus	1.52E+00	3.98E+00	7.14E-05	9.25E-03
Viruses	Schizot4virus	1.50E+00	3.93E+00	8.76E-05	9.73E-03
Viruses	Yuavirus	1.43E+00	2.52E+00	5.62E-04	4.85E-02
Viruses	T4virus	1.42E+00	8.19E+00	8.57E-05	9.73E-03
Viruses	Soymovirus	-2.06E+00	6.88E-01	5.19E-04	4.85E-02
